# Pharms on farms: Variable impacts of veterinary pharmaceuticals on an arbuscular mycorrhizal symbiosis and soil microorganisms

**DOI:** 10.1007/s11104-026-08706-1

**Published:** 2026-06-12

**Authors:** Emily K. Durant, Laura J. Carter, Jill L. Edmondson, Katie J. Field

**Affiliations:** 1 Plants, Photosynthesis and Soil, School of Biosciences,, University of Sheffield, Sheffield, S10 2TN UK; 2 School of Geography, Faculty of Environment, University of Leeds, Leeds, LS2 9JT UK

**Keywords:** Arbuscular mycorrhizal fungi, Doxycycline, Sulfamethoxazole, Flubendazole, Pharmaceutical contamination, Manure

## Abstract

**Background:**

Circular agriculture promotes the reuse of organic waste, such as animal manures, to reduce reliance on synthetic fertilisers. However, manures can introduce veterinary pharmaceuticals into soils. Arbuscular mycorrhizal (AM) fungi are critical for plant nutrition, yet their responses to manure-associated pharmaceuticals remain poorly understood. We investigated the effects of three commonly used veterinary pharmaceuticals—the antibiotics doxycycline (DOX) and sulfamethoxazole (SMX) and the anthelmintic flubendazole (FLUB)—applied at environmentally relevant concentrations, on AM structure and function and wider microbial communities.

**Methods:**

Using dwarf runner bean, we quantified AM-mediated transfer of phosphorus (^33^P) and nitrogen (^15^N) to plants and plant carbon (C) allocation to AM fungi. Bacterial (16S) and fungal (partial 18S and total ITS) community compositions were characterised using DNA amplicon sequencing.

**Results:**

DOX exposure increased AM abundance and AM-mediated ^33^P transfer to plants, and reduced soil bacterial diversity, while plant biomass and N acquisition were unaffected. SMX had no effects on these metrics, despite increasing shoot N content. FLUB reduced AM root colonisation and plant C allocation to AM fungi but did not alter fungal ^33^P or ^15^N transfer or microbial diversity.

**Conclusions:**

Overall, our results demonstrate compound-specific effects of veterinary pharmaceuticals on AM fungal symbioses and soil microbial communities, with DOX positively impacting AM fungi, while FLUB negatively impacted the symbiosis and SMX did not affect AM fungi. DOX reduced soil bacterial diversity. These findings emphasise the importance of considering microbial functional responses when assessing the sustainability and feasibility of manure reuse in agricultural systems.

**Supplementary Information:**

The online version contains supplementary material available at 10.1007/s11104-026-08706-1.

## Introduction

Circular agriculture aims to make food production more sustainable by recycling and applying waste products such as biosolids (treated sewage sludges) and manure to soils to enhance their nutrient status (Rosemarin et al. 2020), thereby creating ‘closed loop’ systems (Kirchherr et al. 2023). Manures have been used to improve soil fertility since at least 5,900 BC (Bogaard et al. 2013) and their application remains a globally widespread practice today (Potter et al. 2010). For example, the European Union (EU-27) and UK produced over 1.4 billion tonnes of animal manure between 2016 and 2019, with over 90% applied to soils (Köninger et al. 2021). These nutrient rich amendments not only reduce waste (Chojnacka et al. 2019), but can also reduce reliance on synthetic fertilisers and have positive impacts on crop yield (Du et al. 2020), positively correlating with soil properties associated with healthy soils (Harris et al. 2023), including soil organic carbon (C) content, available nitrogen (N) content, and bacterial and fungal abundance (Du et al. 2020).

Despite its agronomic benefits, manure application can inadvertently introduce veterinary pharmaceuticals, such as antibiotics and anthelmintics, into soils as treated animals excrete these compounds in their waste (Langhansová et al. 2021; Porto et al. 2021; Albero et al. 2018; Ho et al. 2014; Song And Guo 2014). For example, of the ~110,000 tons of antibiotics used globally to treat livestock in 2019 (Acosta et al. 2025), up to 90% can be excreted unchanged in urine and faeces (Massé et al. 2014). Once introduced into the soil, the persistence and mobility of these pharmaceuticals can vary greatly, depending on factors such as soil type and the pharmaceutical in question, resulting in hard-to-predict effects (Cycoń et al. 2019). These pharmaceuticals can be assimilated into and accumulate in plant tissues (Wei et al. 2023; Carter et al. 2015), causing negative impacts on plant growth and physiology (González-Naranjo et al. 2015; Hillis et al. 2011; An et al. 2009; Liu et al. 2009). For example, the sulfonamide antibiotics sulfamethoxazole and sulfamethazine, commonly prescribed to treat bacterial infections in livestock, have phytotoxic effects on rice (*Oryza sativa* L.), inhibiting seedling shoot and root growth (Liu et al. 2009). These phytotoxic effects could be attributed to direct interactions of the pharmaceuticals with plant roots or indirectly via disruption to soil or root-associated microbial activity. The latter scenario seems particularly likely given activity of soil phosphatase was reduced in treated soils (Liu et al. 2009).

Poultry manure is a nutrient-rich fertiliser (Bolan et al. 2010), high in N and widely applied to soils in regions of intensive poultry production (Lim et al. 2023; Dróżdż et al. 2020; Razali et al. 2018). Commercial poultry are treated with a range of medications, including the antibiotics doxycycline (DOX) and sulfamethoxazole (SMX) for various bacterial infections (Stastny et al. 2023; Gutiérrez et al. 2017). Flubendazole (FLUB) is the only licensed anthelmintic, or de-wormer, for feed addition in the UK (Veterinary Medicines Directorate 2025, accessed 07/08/2025) and is widely used in Europe. DOX has been detected in chicken manure at concentrations between 309–78,516 μg/kg_dry weight (DW)_ (Ho et al. 2014), and SMX at concentrations up to 127 μg/kg_DW_ in chicken manure (Ajibola et al. 2022), while between 86 to 87% of FLUB supplied to poultry is excreted (FAO n.d). As such, there is strong potential for DOX, SMX, and FLUB to enter food production systems via poultry manure application to agricultural soils. However, despite their potential to inadvertently contaminate soils, their potential interactions with soil microbes and plants are unknown, especially as pharmaceuticals only undergo one European regulatory ecotoxicology test on soil microbes (N transformation test) if the need for terrestrial testing is triggered (FERA 2023).

Arbuscular mycorrhizal (AM) fungi represent a key group of soil microbes that can be impacted by pharmaceutical pollution in soil (Durant et al. 2025; Sallach et al. 2021). These globally distributed (Öpik et al. 2013) obligately biotrophic (Bago and Bécard 2002) fungi form symbioses with the roots (or rhizoids/thalli of plants without roots) of around 80% of land plant species (Wang And Qiu 2006), with extraradical hyphae extending beyond the rhizosphere. In soil, AM fungi can improve water retention (Pauwels et al. 2023), reduce the risk of erosion by improving aggregation and aggregate stability (Zhang et al. 2022; Leifheit et al. 2015, 2014; Wu et al. 2014), and bolster soil cycling of N (Veresoglou et al. 2012), P (in partnership with bacteria; Zhang et al. 2018), and C (Parihar et al. 2020). Nutritionally, AM fungi can assist their host plants in their acquisition of soil N and P (Smith And Read 2008) by extending beyond the nutrient depletion zone around roots to access and transfer these otherwise inaccessible nutrients to plant hosts. AM fungi can provide host plants with significant contributions to their P needs (Bago et al. 2000; Douds et al. 2000), even up to just under 100% (Smith et al. 2004), and over 30% of their N needs (Govindarajulu et al. 2005), while receiving their entire C requirements from their host plants (Bago and Bécard 2002). Additionally, AM fungi can facilitate host plant acquisition of water (Püschel et al. 2020) and enhance a plant’s abiotic and biotic stress tolerance (summarised in Thirkell et al. 2017). The benefits AM fungi confer to soils and their host plants form a strong basis for their potential application in agriculture (Schaefer et al. 2021), particularly in terms of enhancing sustainability. This potential is especially relevant in degraded soils (Rog et al. 2025) that are common in agricultural environments (Warui 2024; Environment Agency 2019). In addition to AM fungi, legumes form additional symbioses with N-fixing bacteria called rhizobia, which can substantially contribute to plant N nutrition (Shah et al. 2021). These symbioses can be functionally linked, as N fixation is dependent on plant P status (Zhong et al. 2022), which is often enhanced by AM fungi. Veterinary pharmaceuticals introduced via manure application may therefore affect both AM fungi and rhizobia, potentially altering interacting plant nutrient acquisition pathways.

The extent to which AM fungi confer benefits to their plant hosts is not uniform but instead is highly context dependent. Both biotic interactions, including herbivory (Magkourilou et al. 2024; Durant et al. 2023; Bell et al. 2022; Charters et al. 2020), and abiotic factors, such as atmospheric CO_2_ concentration (Thirkell et al. 2021; Drigo et al. 2013; Field et al. 2012), and pharmaceutical pollution (Durant et al. 2025; Gkimprixi et al. 2023; Sallach et al. 2021; Hillis et al. 2008) can influence the function of AM symbioses. AM fungi can contribute to the degradation of organic pollutants in soil, including pharmaceuticals (Cao et al. 2015), potentially shielding plants from negative impacts. However, the direct contact of soil pharmaceuticals and AM fungi can result in negative impacts on AM fungi. For example, the veterinary anthelmintic albendazole reduced expression of nutrient transporters involved in plant uptake of AM-assimilated soil P and N (Gkimprixi et al. 2023), suggesting that FLUB could have similar impacts. Additionally, exposure to DOX and SMX have been shown to negatively impact AM fungal hyphal length density and spore production in monoxenic carrot root organ cultures (Hillis et al. 2008). The use of poultry manure as a soil amendment therefore represents a plausible pathway through which veterinary pharmaceutical contaminants may interact with, and potentially disrupt, AM fungal communities. Yet it remains unknown whether these compounds influence the functional role of AM fungi in soil-based crop systems, particularly in terms of their capacity to enhance host plant nutrient acquisition or facilitate movement of plant fixed carbon below ground. Disruption of these processes could undermine the agronomic advantages AM fungi could provide within circular agriculture systems. Despite the evidence of negative effects in controlled experimental systems, the impacts of veterinary pharmaceuticals on AM fungal structure and function in soil-based crop systems remain underexplored.

We address this critical knowledge gap by assessing the impacts of DOX, SMX, and FLUB, at environmentally relevant concentrations, on the structure and function (C-for-nutrient exchange between symbionts) of AM associations with the dwarf runner bean (*Phaseolus coccineus* 'Hestia'), a legume also capable of forming symbioses with N-fixing Rhizobia, using radio- and stable-isotope tracers in soil-based experimental systems. To evaluate the impacts of exposure to the veterinary pharmaceuticals on the root and soil microbiome (bacteria and fungi), we used DNA amplicon sequencing to assess community composition and structure. We hypothesized that the veterinary pharmaceuticals tested (DOX, SMX, FLUB) would negatively impact AM colonisation of plant roots, reduce mycorrhiza-derived nutritional benefits for host plants in terms of N and P delivery, and reduce microbial community diversity in line with previous experiments.

## Materials and methods

### Pharmaceutical, crop, and manure selection

Three experiments (referred to as ‘DOX’, ‘SMX’, and ‘FLUB’) were carried out to test the impact of the veterinary pharmaceuticals DOX, SMX, and FLUB on AM structure and function and microbial community composition. Dwarf runner beans (*P. coccineus* cv. 'Hestia') were chosen as our focal crop because they are a dwarf cultivar closely related to the commonly-grown standard non-dwarf runner bean (ICI Business 2022; Edmondson et al. 2020; Schwember et al. 2017). Concentrations of each pharmaceutical were selected from their actual (Ho et al. 2014) or calculated (for SMX and FLUB; see below) concentrations in soil after poultry manure amendment.

### Experimental setup and plant growth conditions

Plants were each grown in 2.65 kg of soil consisting of 1:1 compost:sand (Levington M3 compost (©ICL, UK) and KelKay Horticultural Grit Sand (©The Ames Companies UK Limited)). Compost for FLUB changed to Clover Multi-Purpose Compost (Clover Peat Ltd, Ireland) due to a reformulation (replaced peat with wood fibre; (Lovethegarden n.d) of the Levington M3 compost that resulted in quality deterioration. There was an average of 0.917 ± 0.0323 mg g^−1^ total N, 29.8 ± 1.80 mg g^−1^ total C, and 0.150 ± 0.0109 mg g^−1^ total P in the DOX bulk soil. For SMX, these values were 0.750 ± 0.0361 mg g^−1^, 25.6 ± 1.12 mg g^−1^, and 0.151 ± 0.0114 mg g^−1^. And for FLUB they were 1.65 ± 0.0721 mg g^−1^, 51.3 ± 2.63 mg g^−1^, and 0.201 ± 0.00940 mg g^−1^. There was significantly greater N in the bulk soil used in the FLUB experiment compared to DOX and SMX (p = 0.000248, one-way ANOVA; DOX vs. FLUB: p = 0.00201, SMX vs. FLUB: p = 0.000311, Tukey HSD test). There was also significantly greater C in FLUB soil compared to DOX and SMX (p = 0.00376, one-way ANOVA; DOX vs. FLUB: p = 0.0164, SMX vs. FLUB: p = 0.00469, Tukey HSD test). DOX was planted in February, SMX in June, and FLUB in August, potentially contributing to seasonal growth effects. 35 g/kg_soil_ of a commercially available mycorrhizal fungal inoculum, Rootgrow™ Professional (PlantWorks Ltd, UK), was thoroughly mixed into the bulk soil of each pot. Equal proportions of the following commonly found AM fungi were present in the inoculum: *Funneliformis mosseae, Funneliformis geosporus, Claroideoglomus clarodeum, Rhizophagus irregularis, Glomus micoraggregarum, Diversispora* spp. One ectomycorrhizal fungus (*Scleroderma citrinum*) was also present in the inoculum. The inoculum consisted of a mixture of AM fungal infective propagules (colonised root fragments, hyphae, and spores (approximately 100 per gram)) and an inert carrying substrate (1:1 pumice and zeolite).

In each experiment, soil in half of the pots was spiked with one of the three veterinary pharmaceuticals (‘Drug’ treatment). The soil of the other half of the pots was left un-spiked for an experimental control (‘No Drug’ treatment). Control plants (n = 15) and experimental plants (n = 15) were grown from seed in a glasshouse with a 16-h light day, 60% humidity, 18 °C during the day, and 16 °C at night for 10 weeks. Plants were watered from above as needed, with each pot placed on an individual saucer to collect any runoff and prevent loss of pharmaceuticals through leaching. For DOX, two ‘No Drug’ plants failed to grow (n = 13). For the SMX treatment, two ‘No Drug’ plants and one ‘Drug’ plant failed to grow (n = 13, n = 14, respectively). In each pot, two 35 µm nylon mesh-lined windowed (45 mm long, 25 mm wide) PVC cores (measuring 90 mm long, 40 mm diameter) were filled with bulk soil and inserted into the bulk soil of each pot for nutrient labelling at 8 weeks of growth (see below).

### Pharmaceutical concentration and spiking

Stock solutions were made to a concentration of 1 mg/ml, which were then diluted to make 0.1 µg/µl spiking solutions. Doxycycline hyclate was dissolved in water and SMX and FLUB were dissolved in methanol, as they are insoluble in water. The relevant volumes of each solution were mixed into 25 g of dried KelKay Horticultural Grit Sand per pot. SMX and FLUB spiked sand were dried in a fume hood using N_2_ gas to evaporate the methanol (took approximately 2 h). The same volume of methanol (without the pharmaceuticals) was added to 25 g of unspiked ‘No Drug’ control sands and dried to act as a solvent control. The spiked sand and control sand mixes were thoroughly mixed into the bulk soil of each pot and seeds were sown. Important to note, spiking sand with chemicals is a common procedure (Sallach et al. 2021; Durant et al. 2025) but typically results in immediate increased bioavailability of the pharmaceuticals compared to if they were added in manure (Yang et al. 2026; Al-Wabel et al. 2021). However, the spiked sand was approximately 1.5% of the total sand in the substrate of each pot. Therefore, it was unlikely to alter to bioavailability of the pharmaceuticals when incorporated into the substrate.

*a. Doxycycline (DOX)*

400.00 µg/kg_soil_ doxycycline hyclate (Thermo Scientific Chemicals, ©Thermo Fisher Scientific Inc, Ireland) was added in each ‘Drug’ pot at the time of potting up (see above). Concentrations of between 63.00 and 728.00 µg/kg_soil_ DOX have been previously detected in agricultural soil one month after poultry manure application (10 cm below soil surface; Ho et al. 2014), thus 400.00 µg/kg_soil_ is representative of a mid-level concentration.

*b. Sulfamethoxazole (SMX)*

No concentration of SMX in soil post-poultry manure application was found in a literature search and so a Predicted Environmental Concentration in soil (PEC_soil_), an industry standard metric, was calculated based on Committee for Veterinary Medicinal Products (CVMP) guidelines (Supplementary information; CVMP 2008), resulting in a concentration of 2.89 µg/kg_soil_. D and Ad in equation 1 (Supplementary information) were based on dosage for SMX in the Veterinary Medicines Directorate (Metaxol ®, Veterinary Medicines number 16849/3008).

*c. Flubendazole (FLUB)*

As with SMX, no concentration of FLUB in soil post-poultry manure application was found in a literature search. Therefore, a PEC_soil_ was calculated as above (Supplementary information; CVMP 2008), to yield a FLUB concentration of 70.75 µg/kg_soil_. D and Ad in equation 1 (Supplementary information) were based on dosage for FLUB in the Veterinary Medicines Directorate (Flimabend ®, Veterinary Medicines number 01656/5064).

### ^33^P, ^15^N, and ^14^C labelling

Radio- and stable isotope tracing was performed for all experiments 8 weeks after planting to track C for nutrient exchange between plants and AM fungal symbionts, following the methods of Durant et al. (2025). In each pot, 100 µl solution containing 1 MBq of ^33^P orthophosphate (0.297 ng ^33^P per pot; 111 TBq/mmol Sp Act; Hartmann Analytic, Brunswick, Germany) and 1 mg/ml ^15^N ammonium chloride (0.1 mg ^15^NH_3_Cl per pot; Sigma Aldrich, UK) was injected into one mesh-lined PVC core. Of the 2 mesh-lined PVC cores in each experimental pot, one core remained static (‘static’), and one was gently rotated every 24–48 h after ^33^P and ^15^N injection (‘rotated’) to disrupt AM fungal hyphal connections between the core contents and the host plant. In half of the pots (‘Drug’ treated and ‘No Drug’ controls), the isotopes were added to the static core and in the other half they were added to the rotated core. The addition to rotated cores controls for ^33^P and ^15^N tracers detected in shoot tissues that were accessed via alternative microbial cycling and passive diffusion. By subtracting these values from those detected in plants with access to tracers via intact AM fungal hyphae (where tracers were added to static cores), quantification of ^33^P and ^15^N in plant shoot tissues transferred by AM fungi was calculated.

Two weeks after ^33^P and ^15^N labelling, the PVC cores were capped and cuvettes containing 1 MBq of ^14^C-NaCO_3_ (1.62 GBq/mmol Sp Act; Perkin Elmer, USA) were added to the pots before the headspace of each plant was sealed. Then, 2 ml of 90% lactic acid was injected into each cuvette to liberate a pulse of ^14^CO_2_ gas. Twenty-four hours later, 2 ml 2M KOH was injected into one petri dish per pot and left for 2 h to capture the remaining ^14^CO_2_ before harvest.

### Harvest

Plants were harvested 10 weeks post-germination and shoots and roots were collected. Plants were harvested at the same development stages (budding) across the three experiments. A subsample of roots (approximately 2% of the entire root system) was taken for root staining and AM colonisation assessment. Total bulk soil from each pot was weighed and 2 ~ 10 g subsamples were collected, along with the soils from both ‘static’ and ‘rotated’ PVC cores. Finally, subsamples of roots and bulk soil were collected in Eppendorf tubes and immediately frozen in liquid nitrogen before storage at −80 °C until DNA extraction. Shoots, roots, and bulk soil and core soil subsamples were freeze dried (ScanVac CoolSafe, Labogene, Denmark) after being stored at −20 °C. Further bulk soil subsamples were stored at −20 °C until hyphal length density quantification.

### Root colonisation and hyphal length density

Immediately after harvest, root subsamples (approx. 5% of the total root system) for AM colonisation assessment were placed in histology cassettes and processed as per Vierheilig et al. (1998), beginning with clearing in 10% KOH for 50 min at 70 °C. Next, roots were rinsed with H_2_O and then 1% acetic acid before being placed in acidified ink stain for 24 h to stain. Finally, roots were rinsed in H_2_O again and then placed in 1% acetic acid to remove any residual stain. 20 × 1 cm root segments per plant were mounted on a microscope slide (2 slides per plant) with polyvinyl lacto-glycerol (PVLG) and dried at 70 °C for 48 hr. Finally, the frequency of mycorrhizal colonisation (including all fungal structures) was assessed under 100 × magnification on a light microscope using the magnified intersections method, as per McGonigle et al. (1990).

To quantify the density of AM hyphae in soil, the frozen ~10 g bulk soil subsamples were processed as follows. Briefly, 2–5 g of soil was mixed in 600 ml of H_2_O for 4 min and 200 ml of this was immediately poured into a second beaker. The 200 ml was spun on a magnetic stir plate for 30 s, and 5 s later, a 10 ml sample was taken from 1 cm below the surface with a syringe. This was split into two replicates, and they were passed through a 0.45 µm filter, stained with ink-vinegar, and mounted on microscope slides with PVLG. To prevent soil particles from getting on the filter, the syringes were placed on their sides for 1 min before filtering to allow the soil debris to settle, and the water was ejected carefully. Hyphal length densities were quantified under 100 × magnification using the gridline-intersect method (Brundrett et al. 1994). Unfortunately, one ‘Drug’ sample from the FLUB experiment was missing, so hyphal length densities could not be quantified for that sample (FLUB ‘Drug’ n = 14).

###  Fungal-acquired ^33^P and ^15^N and total P and N analyses

Freeze-dried shoot material was homogenised. For ^33^P analysis, 20–30 mg of each homogenised sample was weighed into acid-washed test tubes in triplicate for acid digestion. 1 ml of concentrated sulphuric acid was added to each tube and left overnight. In the morning, tubes were heated to 350 °C (B5D heat block, Grant Instruments, Cambridge, Ltd.) for 15 min. 100 µl of 35% hydrogen peroxide was then added to each tube and heated for another minute. This step was repeated until solutions became transparent. After cooling, dH_2_O was added to each tube to make up to 10 ml total volume. 2 ml of the diluted digest was added to scintillation vials with 10 ml of the liquid scintillant Emulsify-safe (Perkin Elmer, USA) and ^33^P activity was measured by liquid scintillation counting (Packard Tri-Carb 4910TR, Perkin Elmer, Beaconsfield, UK) and quantified using Eq. 4 (Supplementary information; originally from Cameron et al. 2007). To control for assimilation via diffusion or alternate microbial processes, the amount of ^33^P in shoot tissues in pots where the rotated core was labelled was averaged and this value was subtracted from the amount of ^33^P in shoot tissues where the static core was labelled.

Colorimetry was used to quantify total P in shoot tissues (John 1970; adapted from Murphy And Riley 1962). Briefly, acid-digested samples were mixed with dH_2_O, ascorbic acid, and a developer solution (ammonium molybdate and antimony potassium tartrate reagent) in cuvettes, incubated in the dark for 45 min, and then their optical densities were measured at 882 nm on a spectrophotometer (Jenway® 6300 Visible Spectrophotometer, Cole-Parmer). Total P values were calculated against a standard curve (Supplementary Fig. 1).

To measure total N and ^15^N in shoot tissues for each sample, 3–6 mg of homogenised shoot tissue was weighed into a tin capsule (Sercon, Ltd.) and continuous-flow isotope-ratio mass spectrometry (PDZ 2020 IRMS; Sercon, Ltd.) was conducted. Background activity was quantified using unlabelled dwarf runner bean plant tissue and ^15^N activity in shoot tissues was calculated using Eq. 5 (Supplementary information; Cameron et al. 2006). As with ^33^P, values of ^15^N assimilation in plant tissues where the core containing the isotope was rotated were averaged and then subtracted from ^15^N values of plant tissues where the core containing the isotope remained static.

### Plant-fixed C analyses

30–40 mg of freeze-dried core soils were weighed in duplicate into combusto-cones (Perkin Elmer, USA). Samples were burned by oxidation (307 Packard Sample Oxidiser, Perkin Elmer) and resultant ^14^CO_2_ was captured with 10 ml CarbonTrap (Hidex Chemicals Oy, Finland) and mixed with 10 ml of the scintillant CarbonCount (Hidex Chemicals Oy, Finland) before samples were run on the liquid scintillation counter (Packard Tri-Carb 4910TR, Perkin Elmer, Beaconsfield, UK). Total C (^14^C and ^12^C) content was calculated using Eqs. 6 and 7 (Supplementary information; Cameron et al. 2008). For each pot, the amount of C in rotated core soil was subtracted from that in static core soil to calculate the total plant-fixed C transferred to AM fungi.

### DNA extraction, library preparation, and sequencing

DNA sequencing of soil and root fungi and bacteria was performed to understand how exposure to the three pharmaceuticals tested impacted soil microbial community composition. DNA was extracted from 8 ‘No Drug’ and 8 ‘Drug’ root and soil samples from each of the three experiments. Eppendorf tubes containing 0.1 g root and 0.25 g soil were freeze dried and root samples were homogenised (TissueLyser II, QIAGEN, Venlo, The Netherlands) for 3 min. Then, DNA was extracted following the QIAGEN DNeasy® PowerSoil® Pro Kit instructions. Primary PCRs were carried out on all samples for both bacterial and fungal regions (primers listed in Table 1). 515 F and 806R primers (Caporaso et al. 2011) were used to amplify the 16S region and the following PCR conditions were used: 95 °C for 1 min for denaturation, 95 °C for 30 s, 51 °C for 30 s, 72 °C for 30 s, 30 cycles of annealing, and 72 °C for 10 min for elongation. One DOX ‘No Drug’ root sample contained no 16S DNA and was dropped from further analysis. ITS_F_1624 (Hadziavdic et al. 2014) and R_newITSR (Moeskjær and Hipperson*, in prep*) primers were used to amplify fungal regions (partial 18S and total ITS) of the genome. These primers were used to capture changes in the whole fungal community, rather than AM fungi specifically which would require more specialised primers (Delavaux et al. 2022). The following PCR conditions were used: 95 °C for 15 min for denaturation, 94 °C for 30 s, 55 °C for 1 min, 72 °C for 30 s, 30 cycles of annealing, and 72 °C for 10 min for elongation. After library preparation was performed at the NERC Environmental Omics Facility – Visitor Facility (NEOF-VF; Genomics Laboratory, University of Sheffield, Sheffield, UK), paired-end Illumina sequencing (Illumina, USA) was performed on all root and soil samples by NEOF at the Centre for Genomic Research (University of Liverpool, Liverpool, UK). One 2 × 250 bp Illumina MiSeq v2 was carried out for bacterial sequencing and one 2 × 300 bp Illumina MiSeq v3 was carried out for fungal sequencing (NCBI BioProject ID PRJNA1336642).

**Table 1:** Primary PCR primer names and sequences. Bold = NEOF adapters, underlined = unique molecular identifier, normal text = gene specific primers

Primer	Sequence
515F	**ACACTCTTTCCCTACACGACGCTCTTCCGATCT**-NNNNN-GTGCCAGCMGCCGCGGTAA
806R	**GTGACTGGAGTTCAGACGTGTGCTCTTCCGATCT**-GGACTACHVGGGTWTCTAAT
ITS_F_1624	**ACACTCTTTCCCTACACGACGCTCTTCCGATCT**-NNNNN-CCTTTGTACACACCGCCCGTCG
R_newITSR	**GTGACTGGAGTTCAGACGTGTGCTCTTCCGATCT**-CCAAGAGATCCRTTGYTRAAA

### Read processing and data analysis

Both bacterial and fungal sequences were processed following the paired-end read DADA2 (Callahan et al. 2016) workflow published by Kathryn Maher (https://github.com/khmaher/HPC_dada2), which is largely based on the DADA2 (v.1.8) tutorial published by Benjamin Callahan (https://benjjneb.github.io/dada2/tutorial_1_8.html). Briefly, reads with Ns were first removed, then primer sequences were removed using cutadapt, sequences were trimmed based on quality scores (Bacterial: Forward = 225 bp, Reverse = 175 bp; Fungal: Forward = 225 bp, Reverse = 225 bp), reads were dereplicated, DADA2 denoising was performed, reads were merged, chimeric reads were removed, and finally, taxonomies were assigned to the ASVs. Bacterial ASVs were aligned to the SILVA 138.1 database (Callahan et al. 2016; Yilmaz et al. 2014; Quast et al. 2012) and fungal ASVs to the UNITE 10.0 database (Abarenkov et al. 2025).

Fungal sequences were largely unidentified, likely because the primers used capture the ITS1 region and a partial fragment of the 18S region. To get increased identification, all fungal ASVs were subjected to standard nucleotide BLAST (Camacho et al. 2009; Altschul et al. 1990) and TaxonKit (Shen And Ren 2021) was used to get their taxonomies (reformatted to include only kingdom, phylum, class, order, family, genus, species levels). BLAST code was based on a workflow developed by Ewan Harney, published on https://github.com/ewan-harney/hpc_blast2taxonomizr/tree/main/b2t_scripts (01A and 01B steps), and taxonomic code was developed by the author, Durant, using TaxonKit (Shen And Ren 2021). BLAST results were merged with results from those produced against the UNITE database. Where results were produced from both UNITE and BLAST for the same ASV, the result from UNITE was prioritised. Code for these steps is available in supplementary information.

Once processing was performed, phyloseq objects for roots and soil for fungi and bacteria were made in RStudio version 2024.04.2 (Build 764) (R Core Team 2024; RStudio Team 2020) using the phyloseq package (McMurdie And Holmes 2013) and described using the print_ps() function (microbiomeutilities package; Shetty and Lahti, 2025). They were then filtered using the prune_samples() function (phyloseq package; McMurdie And Holmes 2013) for low read (< 5,000 reads) data and the tax_filter function (microViz package; Barnett et al. 2021) for singletons. DOX and SMX experiments had fungal data from all samples analysed for both root and soil sequencing. Three samples from ‘No Drug’ and one ‘Drug’ sample were removed from FLUB fungal root analyses after filtering for low read data and singletons (‘No Drug’ n = 5, ‘Drug’ n = 7), while all samples were retained for FLUB fungal soil analyses. For bacterial analyses, all root samples were retained after filtering steps for DOX, SMX, and FLUB experiments. For bacterial soil populations, one ‘Drug’ sample was removed from both DOX and SMX experiments (‘No Drug’ n = 8, ‘Drug’ n = 7), while two ‘No Drug’ samples were removed from the FLUB experiment (‘No Drug’ n = 6, ‘Drug’ n = 8) after filtering for low read data and singletons.

Alpha diversity measures (Shannon’s and Simpson diversity indices) were calculated using the estimate_richness() function (phyloseq package; McMurdie And Holmes 2013). Then, phyloseq objects were normalised using the transform_sample_counts() function (phyloseq package; McMurdie And Holmes 2013) and Bray–Curtis distances were computed using the functions distance() and ordinate() (phyloseq package; McMurdie And Holmes 2013). To calculate the mean relative abundances of the top 10 most abundant classes for bacteria in soil, the top 10 most abundant classes overall were identified. This was done by ranking normalised phyloseq objects by class using tax_glom() (phyloseq package; McMurdie And Holmes 2013), converting them to a dataframe using psmelt2() (microbiome package; Lahti And Shetty, 2012–2019), and then using the summarise() function (dplyr package; Wickham et al. 2023) to calculate mean relative abundances of each class and their standard deviations. Lastly, data was ranked in descending order of relative abundance using the arrange() function (dplyr package; Wickham et al. 2023). To investigate changes in mean relative abundances of rhizobial families, taxa belonging to all rhizobial families were retained using the tax_glom(), psmelt(), and filter() functions (phyloseq package; McMurdie And Holmes 2013; dplyr package; Wickham et al. 2023). Then, relative abundances were calculated and summarised by each experiment and treatment before being averaged (dplyr package; Wickham et al. 2023). All data was exported, and graphs were made in GraphPad Prism version 10.4.2 (633) (GraphPad 2025).

### Statistical analyses

All statistical analyses were performed in RStudio (R Core Team 2024; RStudio Team 2020). Welch’s t-tests were performed for plant biomass and nutrition, colonisation, hyphal length density, alpha diversity, and relative abundance data, comparing between treatments of each experiment using the t.test() function (stats package; R Core Team 2024). Data did not satisfy the test assumptions for nutrient exchange metrics; thus Mann–Whitney U tests were performed using the wilcox.test() function (stats package; R Core Team 2024). PERMANOVAs were performed for Bray–Curtis distances (visualised in NMDS plots) using the adonis2() function (vegan package; Oksanen et al. 2024). See supplementary information for R code. All graphs were made in GraphPad Prism version 10.4.2 (633) (GraphPad 2025).

## Results

### Plant biomass and nutrition

**Fig. 1:**
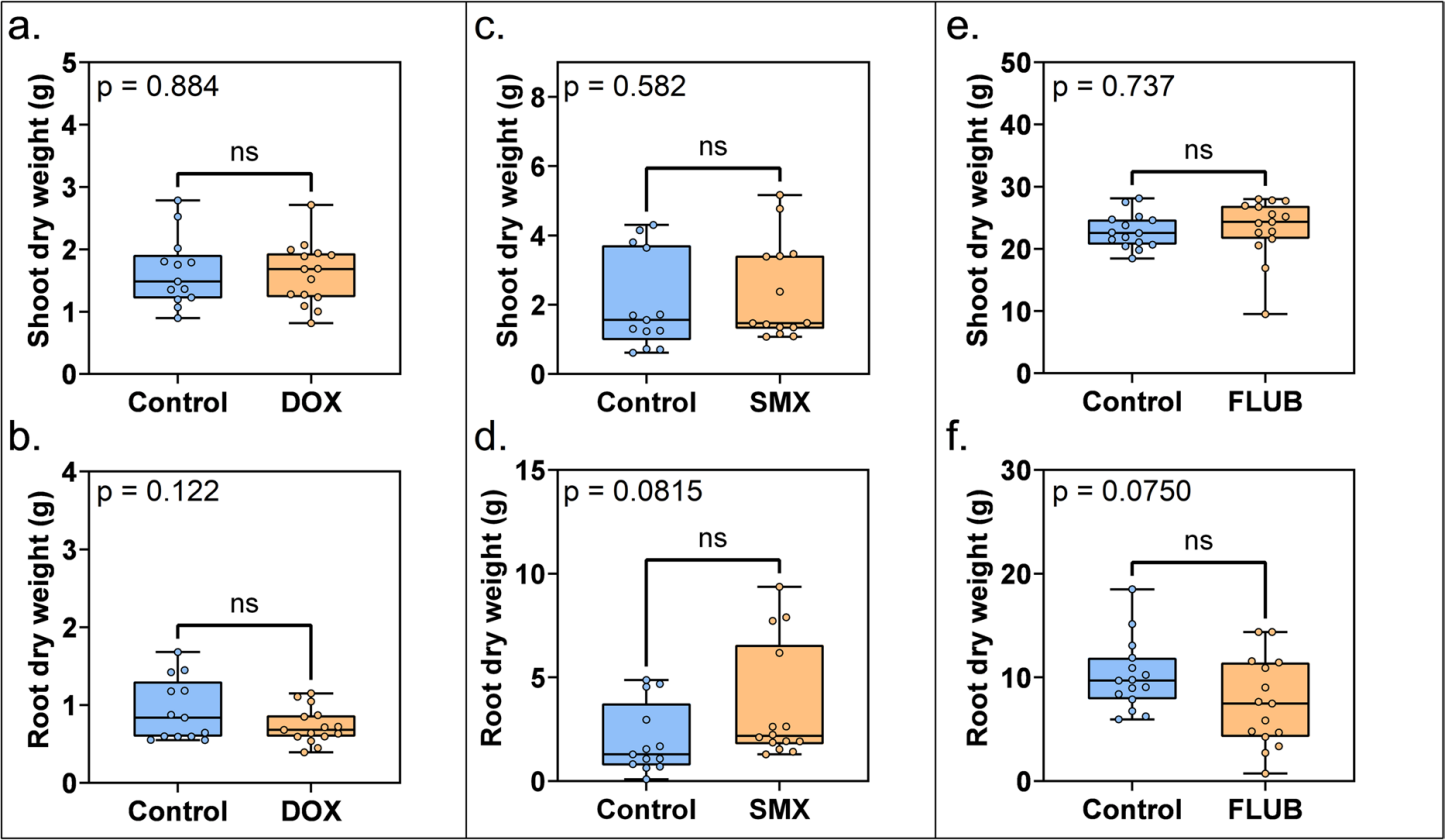
Effects of DOX = a,b; SMX = c,d; and FLUB = e,f on shoot (**a**,**c**,**e**) and root biomass (**b**,**d**,**f**). Control (‘No Drug’) treatments in blue, experimental (‘Drug’) treatments in orange. Whiskers extend to the 25th and 75^th^ percentiles, with boxes encompassing the interquartile range. Median lines exist inside each box. ns = a non-significant difference (*p* > 0.05, Welch’s t-test)

DOX, SMX, and FLUB soil contamination had no effect on shoot or root biomass (Fig. 1; Supplementary Table 1; Welch’s t-test; *p* > 0.05).

**Fig. 2:**
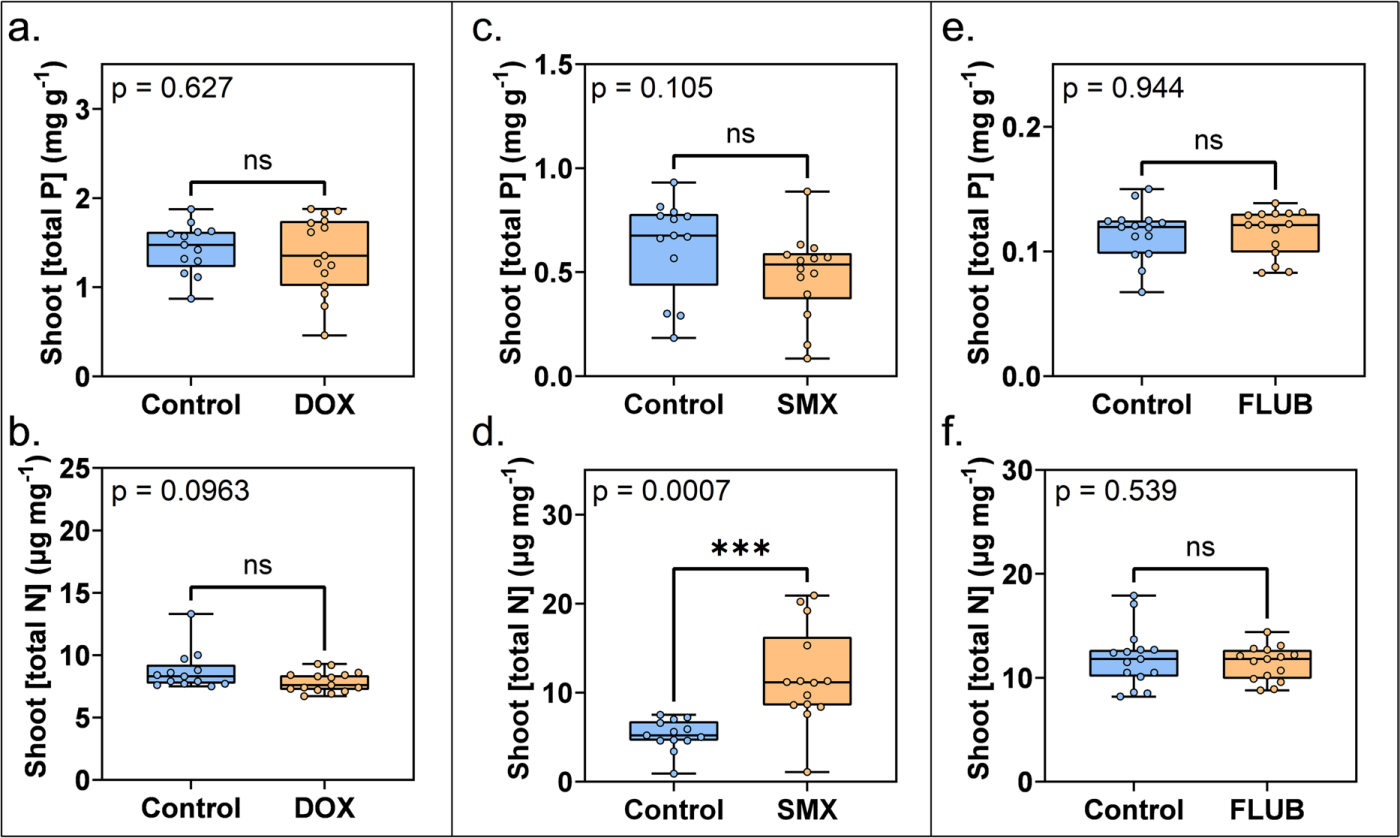
Concentration of P (**a**,**c**,**e**) and N (**b,d,f**) in shoot tissues. DOX = a,b; SMX = c,d; and FLUB = e,f. Control (‘No Drug’) treatments in blue, experimental (‘Drug’) treatments in orange. Whiskers extend to the 25th and 75^th^ percentiles, with boxes encompassing the interquartile range. Median lines exist inside each box. Significant differences (Welch’s t-test) are denoted as *** *p* < 0.001. ns = a non-significant difference (*p* > 0.05)

Similarly, total amount (not normalised to biomass; Supplementary Fig. 2) and concentration of shoot and root P and N (Fig. 2) were unaffected by any of the pharmaceuticals tested (Supplementary Table 2; Welch’s t-test; *p* > 0.05), apart from an increase in absolute and concentration of total N in dwarf runner bean shoots under SMX exposure (Supplementary Table 2; [total N]: *p* < 0.001; total N: *p* < 0.05).

### Impacts of veterinary pharmaceuticals on AM colonisation and extraradical hyphal densities

**Fig. 3:**
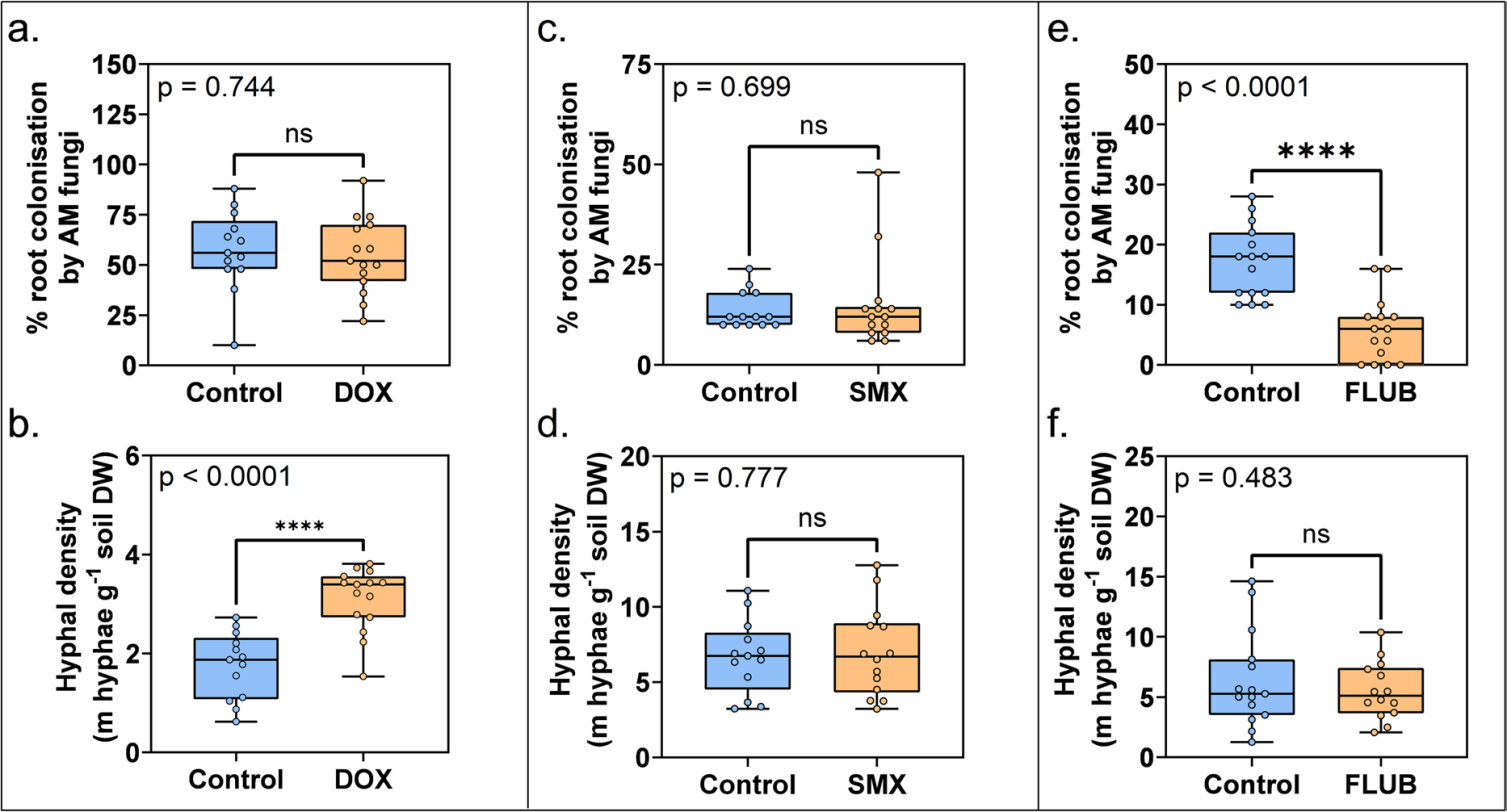
Root colonisation by AM fungi (**a**,**c**,**e**) and soil AM hyphal density (**b**,**d**,**f**). DOX = a,b; SMX = c,d; and FLUB = e,f. Control (‘No Drug’) treatments in blue, experimental (‘Drug’) treatments in orange. Whiskers extend to the 25th and 75^th^ percentiles, with boxes encompassing the interquartile range. Median lines exist inside each box. Significant differences are denoted **** *p* < 0.0001. ns = a non-significant difference (*p* > 0.05, Welch’s t-test)

The effects of the three veterinary pharmaceuticals on root colonisation by AM fungi and AM extraradical hyphal mycelium were variable (Fig. 3; Supplementary Table 3). Although root colonisation by AM fungi was unaffected by exposure to DOX (*p* > 0.05), there was a significant increase in extraradical AM hyphal densities in the surrounding soil (Fig. 3a, b; *p* < 0.0001). AM root colonisation and extraradical hyphal densities were unaffected by the presence of SMX in the soil (Fig. 3c,d; *p* > 0.05). FLUB significantly reduced AM fungal root colonisation (Fig. 3e; *p* < 0.0001) but extraradical hyphal densities were unaffected (Fig. 3f; *p* > 0.05), opposite to the responses observed on exposure to DOX (Fig. 3a, b).

### Differential effects of pharmaceutical exposure on AM fungal-plant nutrient exchange

**Fig. 4:**
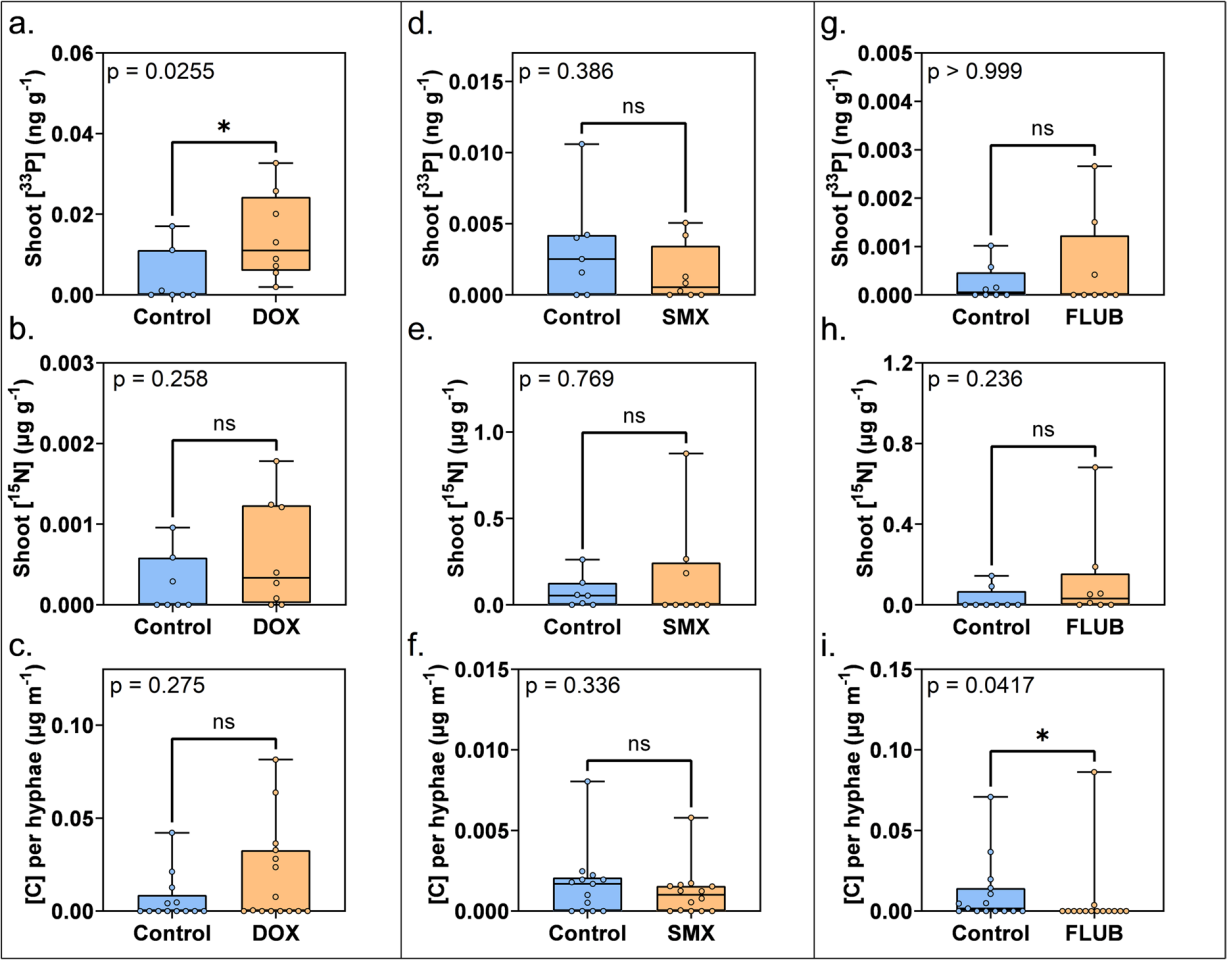
Impact of pharmaceuticals on AM fungal-plant resource exchange. Concentrations of AM-acquired ^33^P and ^15^N in plant shoot tissues (**a**,**b**,**d**,**e**,**g**,**h**). Concentrations of plant-fixed C in extraradical AM fungal hyphae (**c,f,i**). DOX = a,b,c; SMX = d,e,f; and FLUB = g,h,i. Control (‘No Drug’) treatments in blue, experimental (‘Drug’) treatments in orange. Whiskers extend to the 25th and 75th percentiles, with boxes encompassing the interquartile range. Median lines exist inside each box. Significant differences are denoted as * *p* < 0.05. ns = a non-significant difference (*p* > 0.05, Mann–Whitney U test)

The concentration of AM fungal-assimilated ^33^P in plant shoot tissues was significantly greater, by 28.9%, when exposed to DOX (mean Control = 0.00417 ng g^−1^, mean DOX = 0.0144 ng g^−1^; Fig. 4a; Supplementary Table 4; Mann–Whitney U test; *U* = 9, *p* < 0.05), while transfer of ^15^N to host plants and allocation of plant-fixed C to extraradical AM hyphae were unaffected (Fig. 4b,c; Supplementary Table 4; Mann–Whitney U test; *p* > 0.05). C-for-^33^P and -^15^N exchange were not affected by SMX presence in soil (Fig. 4d-f; Supplementary Table 4; Mann–Whitney U test; *p* > 0.05). FLUB also disrupted AM resource exchange dynamics, significantly reducing the concentration of plant-fixed C in extraradical AM hyphae (Fig. 4i; Supplementary Table 4; Mann–Whitney U test; [C]: *U* = 65, *p* < 0.05), despite not impacting AM fungal-acquired ^33^P and ^15^N concentrations in shoots (Fig. 4g,h; Supplementary Table 4; Mann–Whitney U test; *p* > 0.05).

### Impacts on root and soil microbial communities

Exposure to the three pharmaceuticals did not affect fungal community alpha diversity (Shannon and Simpson indices) in roots or soil (Supplementary Fig. 3,4; Supplementary Table 5; Welch’s t-test; *p* > 0.05). Beta diversity (i.e., microbial community composition), visualised by NMDS plots (Supplementary Fig. 6), also did not differ for fungal communities in roots or soil between pharmaceutical contaminated or uncontaminated treatments, regardless of the type of pharmaceutical microbes were exposed to (Supplementary Table 7; PERMANOVA; *p* > 0.05).

**Fig. 5:**
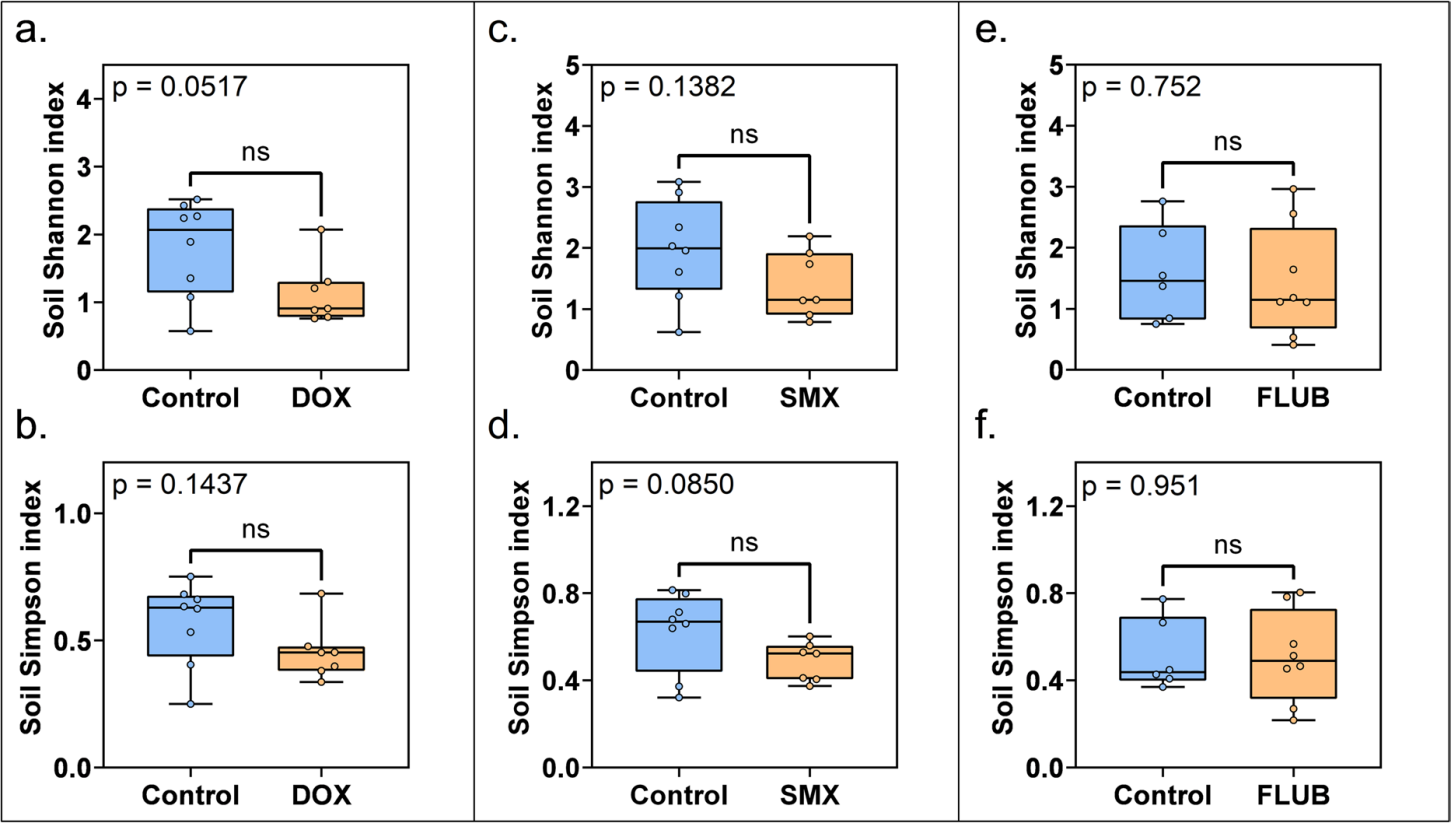
Alpha diversity metrics for bacteria in soil (**a,c,e**: Shannon’s diversity; **b,d,f**: Simpson diversity). DOX = a,b; SMX = c,d; FLUB = e,f. Control (‘No Drug’) treatments in blue, experimental (‘Drug’) treatments in orange. Whiskers extend to the 25th and 75^th^ percentiles, with boxes encompassing the interquartile range. Median lines exist inside each box. ns = a non-significant difference (*p* > 0.05, Welch’s t-test)

There was a trend for reduced Shannon’s bacterial diversity in the soil following DOX treatment (Fig. 5a-d; Supplementary Table 6; Welch’s t-test; Shannnon’s: *p* = 0.0517), but not Simpson (*p* > 0.05) or either alpha diversity metrics in the root (Supplementary Fig. 5a-d; Supplementary Table 6; Welch’s t-test; Shannnon’s: *p* > 0.05). Bacterial alpha diversities in root and soil were unaffected by SMX and FLUB contamination (Supplementary Fig. 5e,f; Fig. 5e,f; Supplementary Table 6; Welch’s t-test; *p* > 0.05). As with fungal communities, beta diversity (Supplementary Fig. 7) did not differ for bacterial communities in roots or soil between control and experimental treatments for DOX, SMX, or FLUB (Supplementary Table 7; PERMANOVA; *p* > 0.05).

**Fig. 6:**
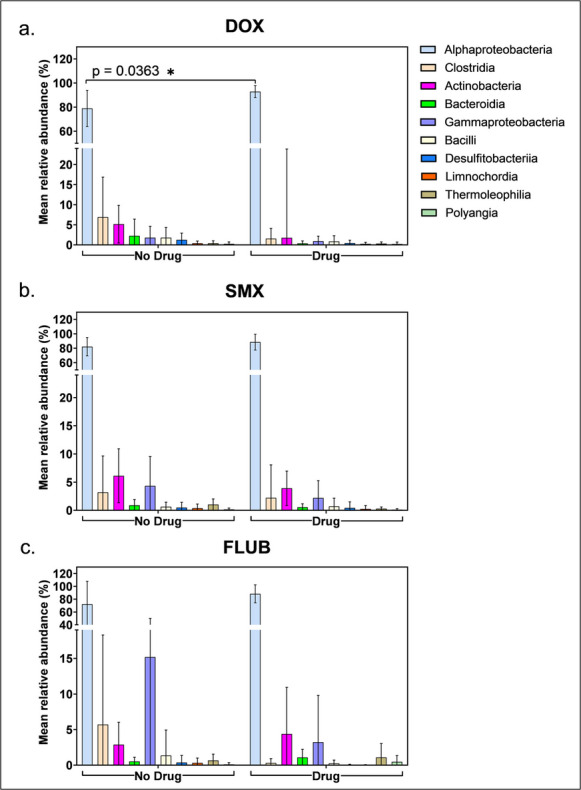
Mean relative abundance of the top 10 most abundant bacterial classes in soil for DOX (**a**), SMX (**b**), and FLUB (**c**). Bacterial classes are coloured as pictured in the key, in the same order as they are listed in the key. Error bars represent standard deviation. Significant differences are denoted as * *p* < 0.05 (Welch’s t-test). All other comparisons between the relative abundance of each class under control vs. experimental treatment are non-significant

To understand the changes in bacterial classes in soil between control and experimental treatments, the mean relative abundances of the top 10 most abundant classes were plotted (Fig. 6). *Alphaproteobacteria* mean relative abundance significantly increased under DOX treatment (Supplementary Table 7; Welch’s t-test; *p* < 0.05), while other classes were unaffected (Supplementary Table 7; Welch’s t-test; *p* > 0.05). There were no changes under SMX or FLUB exposure (Supplementary Table 7; Welch’s t-test; *p* > 0.05). We further plotted the mean relative abundances of the rhizobial family Rhizobiaceae in soil and roots for each experiment (Supplementary Fig. 8). Only Rhizobiaceae and Hyphomicrobiaceae were present, but the latter had negligible relative abundance, always under 0.17% for soil and 0.7% for roots. There was no change in mean relative abundance of Rhizobiaceae when exposed to either DOX, SMX, or FLUB compared to the control (Supplementary Fig. 8; supplementary Table 9; Welch’s t-test;* p* > 0.05).

## Discussion

Governments are increasingly promoting resource reuse in agriculture as a means of reducing waste production (DEFRA 2020); Schroeder et al. 2019). The application of biosolids and manures to agricultural soils is generally considered to be beneficial as it increases soil organic matter content (Liu et al. 2020; Albiach et al. 2001), improves soil structure (Albiach et al. 2001), and supports beneficial soil microbial communities (Liu et al. 2020), which could lead to long-term gains in crop production (Du et al. 2020). However, application of manures in this way may also introduce persistent veterinary pharmaceuticals, both prescribed and unregulated, to the soil (Albero et al. 2018; Ho et al. 2014; Song And Guo 2014). Our results show such compounds can have specific effects on AM fungal structure, plant nutritional gains via AM fungi, and root and soil microbial community composition and structure.

Overall, DOX had more impacts on the variables measured compared to SMX and FLUB. While plant biomass, nutritional status (shoot P and N), and root colonisation by AM fungi were unaffected by DOX exposure, extraradical AM hyphal networks were larger in DOX-contaminated soils. This response may be linked to the observed reduction in soil bacterial diversity in our experimental systems when exposed to DOX, potentially reflecting decreased bacterial abundance in the presence of an antibiotic (Yan et al. 2018). Although commercial potting soil was used in this study, the bacterial classes we identified belong to phyla that are commonly found in many agricultural soils (Lee et al. 2020). Both bacteria and AM fungi rely on plant-derived C from their plant hosts, either through direct transfer in the case of AM fungi or indirectly via plant root exudates for bacteria (Dennis et al. 2010), which both come from the same C stock in the plant. Consequently, potential reductions in bacterial populations may have increased the availability of plant C available to AM fungi, stimulating increased AM fungal hyphal growth. Alternatively, potential reductions in bacterial populations could have resulted in reduced bacterial signalling that causes rhizodeposition by plants (Nguyen 2009), allowing more C to be transferred to AM fungi. A third hypothesis exists, too –DOX may have reduced populations of bacteria that are antagonistic to AM fungi (Miransari, 2011), resulting in increased AM fungal populations. These findings contrast with results in Hillis et al. (2008), who observed reduced colonisation and hyphal densities when exposed to DOX concentrations as low as 10 µg/L. However, that study was conducted in a sterile, agar-based environment using carrot root organ cultures, in which AM fungi and roots were in direct contact with DOX, potentially amplifying any toxic effects. Additionally, bacterial degradation of DOX, which occurs in soil, would have been absent, lengthening the half-life of the chemical (Yan et al. 2018). In soil systems, such as employed in this study, such an interaction is likely to be more limited as DOX strongly sorbs to soil particles (Xu et al. 2021), reducing its bioavailability (Wang And Wang 2015) and limiting direct toxic interactions. This sorption process in soil is governed by properties such as pH, cation exchange capacity (the ability of soil to hold onto positive ions), particle size, organic matter content, and soil type (Thompson And Goyne 2012).

In terms of AM function, ^33^P transfer from AM fungi to plant hosts increased under DOX exposure, possibly reflecting the expansion of extraradical hyphal networks observed in contaminated soils. Larger AM hyphal networks can enhance nutrient foraging capacity (Jansa et al. 2003), allowing greater assimilation and transfer of ^33^P to plant hosts. This aspect of the symbiosis is likely less pronounced than it would be in field soils as pot systems tend to have limited soil without roots accessing their own nutrients. This response appears to be nutrient specific, as ^15^N transfer was unaffected by exposure to DOX. As the plants tested here were legumes and the soil was unsterilised, the plants had multiple routes by which they could acquire N, including direct root uptake, associations with free-living soil bacteria, symbiotic rhizobia (root nodules were observed but not quantified), and AM fungi. Therefore, plant demand for symbiotically fixed N may have been fulfilled by alternative bacterial N-fixation rather than AM-acquired ^15^N under both control and DOX treatments. Consistent with this interpretation, bacterial taxa within the *Alphaproteobacteria* class increased in relative abundance when exposed to DOX in our experiments. This class includes groups involved in N-fixation and other N-cycling processes, including rhizobia (Tsoy et al. 2016), although the relative abundances of rhizobial families (Rhizobiaceae and Hyphomicrobiaceae) were unchanged. This suggests that rhizobial communities may be resilient to DOX exposure, maintaining N transfer to host plants despite wider shifts in *Alphaproteobacteria*. As such, AM fungal-mediated N was likely less in demand by the plants than it might be in non-leguminous species. This functional redundancy may explain why DOX-driven changes in structure and ^33^P transfer were not accompanied by changes in ^15^N transfer. Some caution should be taken when interpreting these results, however, as it is difficult to infer bacterial function from class level phylogenetics (Zhu et al. 2015) and relative abundance does not equal absolute abundance. Collectively, these findings emphasise the need for more research to understand how pharmaceutical contaminants impact soil microbial community diversity and to disentangle the responses of multiple symbiotic pathways. They also highlight the importance of adopting a conservative approach to the introduction of such contaminants into agricultural soils, given the complexity of soil systems and the difficulty of predicting ecological outcomes where many interacting organisms are involved (Anthony et al. 2023; Gardi And Jeffery 2009; Lavelle et al. 2006).

Interestingly, while AM ^33^P transfer to host plants was affected by DOX exposure, plant-fixed C assimilation by AM fungi appeared unaffected. This discrepancy may reflect methodological differences in tracer integration times. C assimilation was quantified after only 24 h of ^14^C labelling, which is short compared to the two-weeks labelling periods used for ^33^P and ^15^N labelling. Although this duration is likely sufficient to incorporate ^14^C into plant sugars, it is unlikely to capture C allocation to compounds involved in fatty acid synthesis and transfer to AM fungi, thus limiting the accurate quantification of C assimilation by AM fungi to recent photosynthates only (Bell et al. 2024). In addition, resource exchange between mycorrhizal symbionts is not necessarily tightly coupled when external pressures are applied to the symbiotic system (Durant et al. 2023; Bell et al. 2022; Charters et al. 2020). For example, a change in soil bacterial populations could have knock-on effects for plant C utilisation, such as changes in immune and stress responses or hormone balance (Pantigoso et al. 2022), which could result in less C available to allocate to AM fungi by host plants.

In contrast to DOX, the second antibiotic tested (SMX) had no detectable impacts on AM symbiosis or on root- and soil-associated fungal and bacterial communities, likely due to the low concentration used in this study. SMX is typically found in manure amended soil at substantially lower concentrations than DOX—2.89 µg/kg_soil (DW)_ vs. 400 µg/kg_soil (DW)_. Although Hillis et al. (2008) reported negative effects of SMX on AM colonisation and extraradical hyphal density, effects were observed to occur at higher concentrations—45 µg/L—and in monoxenic conditions without the potential for soil bacterial degradation and sorption to soil particles – factors that would otherwise limit bioavailability. Interestingly, shoot total N (content and concentration) increased under SMX exposure, whereas shoot total P was unaffected. Given that there were no changes in soil bacterial diversity, in contrast with DOX, it is unlikely that increased total N is due to root-associated, N-fixing bacteria. This interpretation is further supported by the absence of phylum-level changes in relative abundance of N-fixing bacterial taxa or relative abundances of rhizobial families, though it should be noted that relative abundance does not necessarily reflect absolute abundance. However, as rhizobial N fixation activity was not directly measured, this cannot be excluded. Given the large role of rhizobial in supplying legumes with N (Shah et al. 2021), subtle changes in their activity could influence plant N status without changes in relative abundance. Direct assimilation of nitrogen from SMX by plants is also unlikely, as the compound contains only three N atoms per molecular and was present in this system at very low concentrations. Finally, as the same soil was used in both control and experiment treatments, the increase in shoot total N is not likely to have been a result of high soil P content, which can stimulate plant N assimilation via increased microbial activity (Xia et al. 2023).

The benzimidazole-type anthelmintic FLUB had no detectable effects on plant metrics or root- or soil-associated microbes. AM root colonisation was reduced when exposed to FLUB, although no changes in fungal diversity were observed via DNA sequencing. Reduced colonisation is consistent with findings from studies investigating albendazole (Gkimprixi et al. 2023), another benzimidazole, where the proposed mechanism of action of inhibiting microtubule formation during mitosis in worms might also occur in AM fungal cells. As FLUB shares this broad mechanism of action (Lacey 1990), it may similarly impair AM fungal growth and colonisation, providing further evidence that benzimidazoles can exert off-target effects on non-target organisms. Although some benzimidazoles are used as fungicides, FLUB was not designed to be one. However, a model predicted that it could bind to β-tubulin in the cryptococcal meningitis fungus, and antifungal activity was observed in mice (Nixon et al. 2018). Despite reduced colonisation observed here, transfer of ^33^P and ^15^N from AM fungi to plant hosts was unaffected by exposure to FLUB, indicating that colonisation intensity is not always directly proportional to mycorrhizal resource transfer (Frew 2025). This is particularly relevant given that AM fungi can confer many additional, non-nutritional benefits to host plants (Püschel et al. 2020; summarised in Thirkell et al. 2017). In contrast, plant-fixed C assimilated by AM fungal hyphae was reduced in the presence of FLUB, likely reflecting the diminished extent of root colonisation by obligately biotrophic AM fungi.

Considering these results together, it is clear that there are context-specific impacts of manure-associated soil contaminant exposure on plant and soil microbial communities. The extent and type of effects of exposure to the compounds tested here were largely dependent on the type of veterinary pharmaceutical present in the soil. Specifically, DOX and FLUB, but not SMX, can alter AM fungal structure and aspects of plant-AM fungal resource exchange. While exposure to DOX was associated with apparently beneficial responses for AM fungi and plants, including expanded hyphal networks and enhanced AM fungal-acquired ^33^P transfer to host plants, concurrent reductions in soil bacterial diversity highlight the importance of adopting a whole-community perspective when assessing contaminant impacts. Focusing on a single microbial group in isolation risks overlooking trade-offs and indirect effects that emerge only when multiple components of the soil microbiome are considered. For instance, pharmaceutical-induced shifts in bacterial communities could indirectly influence key soil processes such as N fixation, organic N mineralisation, or plant N uptake, thereby affecting plant–microbe interactions in ways that are difficult to detect when examining individual microbial groups alone. A key limitation of this study is that it focused on AM fungi, although legumes also form symbioses with N-fixing rhizobia. Future work should incorporate both AM fungi and rhizobia into experiments to disentangle how veterinary pharmaceuticals influence the dynamics between the symbionts and their intertwined relationship with legumes. Investigating microbial activity and community responses using approaches such as functional assays or omics-based analyses would also help clarify potential indirect pathways through which pharmaceuticals affect soil microbial processes. In terms of future food security, the absence of treatment effects on plant biomass suggests that, under the conditions tested, these contaminants may have limited short-term impacts on yield. While shoot biomass can be linked to crop yield, it is not always in *Phaseolus* species (AraújoI and Teixeira Araújo 2008), therefore, future studies linking veterinary pharmaceutical contamination to crop yield are warranted.

It is also important to note that our experiments tested the effects of single contaminants in isolation. In reality, manure contains mixtures of pharmaceuticals (Carter et al. 2025; Wohde et al. 2016), whose combined effects may be additive or synergistic (Boxall 2004). However, it remains important to gain initial mechanistic insights on single compound exposure before moving to more complex field-scale or pharmaceutical mixture studies. Additionally, the responses observed in this experiment reflect short-term exposure to veterinary pharmaceuticals, similar to what might occur following manure application to soil. Future studies examining whether soil microbes adapt to longer-term exposure or to the accumulation of these contaminants would also provide valuable insights. There is also evidence that fungi can break down organic pollutants in soil through mineralisation, however little work has been done on the role of AM fungi in this (Lenoir et al. 2016). It would therefore be interesting to study their role in the breakdown of pharmaceuticals. Furthermore, many countries recommended storing and/or composting solid poultry manure prior to application to soils, as this process can reduce pharmaceutical concentrations (Dolliver et al. 2008). For example, the UK government recommends storing poultry manure to mitigate nutrient pollution and parasite risks (DEFRA et al. 2009). However, farmers are not legally required to follow this advice. As our understanding of how manure storage reduces pharmaceutical concentrations improves, introducing requirements for manure storage could be a worthwhile policy consideration. Given these uncertainties and potential for unintended ecological consequences, reducing the use of pharmaceuticals in livestock and poultry, improving manure treatment processes, and advancing research into novel effective decontamination strategies are critical steps to safeguard soil health, agricultural sustainability, and long-term food security.

## Supplementary Information

Below is the link to the electronic supplementary material.

Supplementary file1 (DOCX 152 KB)

## Data Availability

The datasets generated during and/or analysed during the current study are available from the corresponding author on reasonable request. DNA sequence datasets are available from the National Center for Biotechnology Information (NCBI) GenBank under BioProject ID PRJNA1336642 (http://www.ncbi.nlm.nih.gov/bioproject/1336642).
