# Supplementary material for: Pharms on farms: Variable impacts of veterinary pharmaceuticals on an arbuscular mycorrhizal symbiosis and soil microorganisms
Source: Plant Soil. 2026 Jun 12;524(2):1077–97. doi: 10.1007/s11104-026-08706-1 (PMC13437658; doi:10.1007/s11104-026-08706-1)
Supplement: Supplementary file 1 [file 11104_2026_8706_MOESM1_ESM.docx]

*Research Article*

**Pharms on farms: Variable impacts of veterinary pharmaceuticals on an arbuscular mycorrhizal symbiosis and soil microorganisms**

Emily K. Durant^1^, Laura Carter^2^, Jill Edmondson^1^, Katie J. Field^1*^

*^1^Plants, Photosynthesis and Soil, School of Biosciences, University of Sheffield, Sheffield, UK S10 2TN*

*^2^School of Geography, Faculty of Environment, University of Leeds, Leeds, UK LS2 9JT*

*Corresponding author: k.j.field@sheffield.ac.uk

ORCID’s: Emily K. Durant [(0009-0004-6696-204X](https://orcid.org/0009-0004-6696-204X)), Laura Carter (0000-0002-1146-7920), Jill Edmondson (0000-0002-3623-4816), Katie J. Field (0000-0002-5196-2360).

**Key words:**

Arbuscular mycorrhizal fungi, doxycycline, sulfamethoxazole, flubendazole, pharmaceutical contamination, manure

**Acknowledgements**

We thank Dr. Alex Williams, Dr. Besiana Sinanaj, Dr. Nathan Howard, Dr. Emily Magkourilou, and Dr. Miles Bate-Weldon for assistance harvesting experiments. DNA sequencing was funded by the Natural Environment Research Council Environmental Omics Facility (NEOF1648). E.K.D. is supported by an Ecotoxicological Risk Assessment Towards Sustainable Chemical Use (ECORISC) PhD (NE/V013041/1) and we thank the de Laszlo Foundation for supporting PhD student research.

**Materials and methods**

*Pharmaceutical concentration and spiking*

**Equation 1:** $Mi=D x Ad x BW x Fh$

**Equation 2**: $Mt=Mi x e^{(\frac{-\ln\left( 2 \right)x\left( \frac{Tst}{2} \right)}{{DT}_{50}})}$

**Equation 3**: ${PEC}_{soil refined}=\left( \frac{Mt x 170}{(1500 x 10000 x 0.05 x Ns} \right) x 1000$

where:

PEC_soil refined_ = The refined Predicted Environmental Concentration in soil [µg/kg]

Mi = Mass of active in manure/slurry [mg]

D = Daily dose of the active ingredient [mg/kg_bw_ / d]

Ad = Number of days of treatment [d]

BW = Animal body weight [kg_bw_] (see Table 6).

Fh = Fraction of the herd treated [value between 0 and 1]

Tst = Length of time manure is stored [days]

DT_50_ = Half-life of active in manure [days]

Mt = Mass of active in manure/slurry after the mean storage time [mg]

170 = EU nitrogen spreading limit [kg.N/ha]

1500 = Bulk density of dry soil [kg/m^3^]

10000 = Area of 1 hectare [m^2^/ha]

0.05 = Depth of penetration into soil [m]

Ns = Nitrogen produced during storage time [kgN]

1000 = Conversion factor [1000µg/mg]

*Fungal-acquired ^33^P and ^15^N and total P and N analyses*

**Equation 4:** M^33^P = $\left( \frac{\left[ \frac{cDPM}{60} \right]}{SAct} \right)MwtDF$

Where M^33^P = mass of ^33^P (mg); cDPM = counts as disintegrations per minute; SAct = specific activity of the course (Bq mmol-1); DF = dilution factor; and Mwt = molecular mass of P.

**Supplementary fig 1 Standard curves for DOX (a.), SMX (b.), and FLUB (c.) total P calculations.** A linear regression line was fitted for each graph and its equation and R^2^ value are written on the graphs for each experiment.

**Equation 5:** $M_{Ex}= \left( \frac{\left[ {At}_{lab} - {At}_{cont} \right]}{100} \right)\left( M\left[ \frac{\%N}{100} \right] \right)$

Where MEx = mass excess of the isotope (g); Atlab = atom percentage of the isotope in labelled microcosm; Atcont = atom percentage of the isotope in paired control microcosm; M = biomass of sample (g); and % N = percentage of the nitrogen.

*Plant-fixed C analyses*

**Equation 6:** T_pf_ or T_pa_ = $\left( \left[ \frac{A}{A_{sp}} \right]m_{a} \right)+ \left( P_{r} \times m_{c} \right)$

Where T_pf_ or T_pa_ = total C transferred from plant to fungus (g); A = radioactivity of the tissue sample (Bq); A_sp_ = specific activity of the source (Bq Mol^-1^); m_a_ = atomic mass of ^14^C; P_r_ = proportion of the total ^14^C label supplied present in the tissue; and m_c_ = mass of C (g) in the CO_2_ present in the labelling chamber, from the ideal gas law (Equation 7).

**Equation 7:** M_cd_ = $M_{cd}\left( \frac{PVcd}{RT} \right)\mathrm{thus} m_{c} = m_{cd} \times0.27292$

Where m_cd_ = mass of CO_2_ (g); M_cd_ = molecular mass of CO_2_ (44.01 g mol^-1^); P = pressure (kPa); V_cd_ = volume of CO_2_ in the chamber (0.003m3); m_c_ = mass of unlabelled C in the labelling chamber (g); M= Molar mass (12.011 g); R = universal gas constant (J K^-1^ mol^-1^); T = absolute temperature (K); m_c_ = mass of C (g) in the CO_2_ present in the labelling chamber, where 0.27292 is the proportion of C in CO_2_ on a mass fraction basis.

*Read processing and data analysis*

Read processing was performed using HPC Bessemer at the University of Sheffield. Code used for fungal BLAST steps was developed based on the code developed by Ewan Harney (<https://github.com/ewan-harney/hpc_blast2taxonomizr/tree/main/b2t_scripts>). Then, the author Emily Durant developed the code for taxonomy identification.

**Subject all sequences to BLAST:**

1. In your HPC directory, make project folder and logs folder.

mkdir /fastdata/bop22ekd/exp1-3/ITS_unknown_taxonomy

This is my project directory folder

mkdir /fastdata/bop22ekd/exp1-3/ITS_unknown_taxonomy/logs

- This ‘logs’ folder will store log files
  2. Create a fasta file with all sequences and their ASV numbers and move this into your ITS_unknown_taxonomy folder. Do this for all sequences, even ones that had known taxonomies. Therefore, I am using the 06_ASV_seqs_Copy.fasta file from the dada2 pipeline.
  3. Cut the 06_ASV_seqs file into smaller files containing 100 sequences to make BLAST’ing faster.

qsub 01_run_blast_prep.sh split_fasta 06_ASV_seqs_Copy.fasta

split_fasta = file directory name where the smaller files will be sent. The file names will be called split_fasta_list.txt

06_ASV_seqs_Copy.fasta = the path to my data (I am in the ITS_unknown_taxonomy folder when running this code).

4. Check this worked by counting the number of smaller files that were created.

wc -l split_fasta_list.txt

5. BLAST sequences in smaller files.

sbatch --array=1-41 02_run_split_blast_array.sh split_fasta blast_out

1-41 = the number of files you have

sbatch --array=1-41 02_run_split_blast_array.sh = Launches the script as an array, running 41 different subjobs (one for each file)

split_fasta = the input directory will the smaller files

blast_out = the output directory where the results go to

6. Concatenate files.

cat blast_out/chunk*.fa.out.tab > blast_SSU_out_complete.txt

blast_SSU_out_complete.txt = output file name

**Scripts needed for subjecting sequences to BLAST:**

**Script_01:**

#!/bin/bash

#SBATCH --job-name=blast_prep

#SBATCH --output=blast_prep

#SBATCH --nodes=1

#SBATCH --ntasks-per-node=1

#SBATCH --cpus-per-task=1

#SBATCH -A molecolb

#SBATCH -p molecolb

#SBATCH -o logs/blast_prep_%a.out # Standard output

#SBATCH -e logs/blast_prep_%a.err # Standard error

#SBATCH --mem-per-cpu=1GB

#SBATCH --time=24:00:00

#SBATCH --mail-type=END

#SBATCH --mail-user=e.durant@sheffield.ac.uk

\## load profile

source ~/.bash_profile

conda activate /usr/local/extras/Genomics/apps/mambaforge/envs/metabarcoding

MAIN_DIR=$PWD

PREP_DIR=$1

FASTA_PATH=$2

mkdir -p ${MAIN_DIR}/${PREP_DIR}

cd ${MAIN_DIR}/${PREP_DIR}

awk 'BEGIN {n=0;file=sprintf(C"chunk%d.fa", n)} /^>/ {if(seq_count % 100==0 && seq_count !=0) {n++; file=sprintf("chunk%d.fa",n);} seq_count++;} { print > file; n++; next;} { print >> file; }' < ${MAIN_DIR}/${FASTA_PATH}

ls chunk* > ${MAIN_DIR}/${PREP_DIR}_list.txt

**Script_02:**

#!/bin/bash

#SBATCH --job-name=blast_array

#SBATCH --output=blast_array

#SBATCH --nodes=1

#SBATCH --ntasks-per-node=1

#SBATCH --cpus-per-task=5

#SBATCH -A molecolb

#SBATCH -p molecolb

#SBATCH -o logs/blast_array_%a.out # Standard output

#SBATCH -e logs/blast_array_%a.err # Standard error

#SBATCH --mem-per-cpu=5GB

#SBATCH --time=6-00:00:00

#SBATCH --mail-type=END

#SBATCH --mail-user=e.durant@sheffield.ac.uk

\## load profile

source ~/.bash_profile

conda activate /usr/local/extras/Genomics/apps/mambaforge/envs/metabarcoding

MAIN_DIR=$PWD

PREP_DIR=$1

OUT_DIR=$2

ncbi_nt=/usr/local/extras/Genomics/db/ncbi_nt/current/nt

mkdir -p ${MAIN_DIR}/${OUT_DIR}

data=$(sed "${SLURM_ARRAY_TASK_ID}q;d" <(cat ${PREP_DIR}_list.txt))

fasta_file=$(echo "$data" | cut -f1 )

blastn -query ${MAIN_DIR}/${PREP_DIR}/${fasta_file} \

-task blastn \

-db ${ncbi_nt} \

-out ${OUT_DIR}/${fasta_file}.out.tab \

-num_threads 4 \

-outfmt "7 qseqid sseqid pident length mismatch gapopen qstart qend sstart send evalue bitscore qseq staxids"

**Retrieve taxonomic information from BLAST results:**

1. Install taxonkit

conda install -c bioconda taxonkit

2. Extract TaxIDs for blast_SSU_out_complete.txt file (in 14^th^ column): In ITS_unknown_taxonomy folder:

cut -f 14 blast_SSU_out_complete.txt > taxids.txt

3. Download NCBI taxonomy database

mkdir -p ~/.taxonkit # Create taxonkit directory if not exists

cd ~/.taxonkit

wget ftp://ftp.ncbi.nih.gov/pub/taxonomy/taxdump.tar.gz # Download taxonomy dump

tar -xzf taxdump.tar.gz names.dmp nodes.dmp delnodes.dmp merged.dmp # Extract required files

4. Verify the files. You should see: names.dmp, delnodes.dmp, and merged.dmp

ls ~/.taxonkit

5. Because my taxids.txt file is huge, I needed to break it into smaller files. Process TaxIDs in smaller chunks

split -l 1000 taxids.txt taxids_part_

6. Use taxonkit to get taxonomic info for each smaller file. Wrapping the lineage output in quotes makes it so any taxonomic names containing spaces are treated as a single field (aka not separated into different columns)

for file in taxids_part_*; do

taxonkit lineage "$file" | awk -F'\t' 'BEGIN {OFS="\t"} {print $1, "\"" $2 "\""}' > "${file}_lineage.txt"

done

7. Use reformat function to extract taxonomic ranks of interest

for file in taxids_part_*_lineage.txt; do

awk -F'\t' 'BEGIN {OFS="\t"} {gsub(/"/, "", $2); print $1, $2}' "$file" | taxonkit reformat -f "{k};{p};{c};{o};{f};{g};{s}" > "${file}_filtered.txt"

done

8. Job was killed midway through because time expired 🡪 Find taxid lineage files that weren’t processed. Since the taxonkit reformat output results as *lineage.txt_filtered.txt, you can check with original taxid lineage files **don’t have a corresponding output file**:

for file in taxids_part_*_lineage.txt; do

if [[ ! -f "${file}_filtered.txt" ]]; then

echo "$file"

fi

done > missing_files.txt

9. Have list of missing files, so now reprocess them:

while read file; do

awk -F'\t' 'BEGIN {OFS="\t"} {gsub(/"/, "", $2); print $1, $2}' "$file" | taxonkit reformat -f "{k};{p};{c};{o};{f};{g};{s}" > "${file}_filtered.txt"

done < missing_files.txt

10. Merge results

cat taxids_part_*_lineage.txt_filtered.txt > full_taxonomy_results.txt

11. Check merge looks good

head full_taxonomy_results.txt

wc -l full_taxonomy_results.txt # Check total number of lines

12. Some names have spaces in them (like ‘uncultured fungus’) and this is making it so they are split into different columns here. I need to get rid of this – do it was replacing those spaces with an underscore

awk -F'\t' 'BEGIN {OFS="\t"} {for (i=2; i<=NF; i++) gsub(/ /, "_", $i); print}' full_taxonomy_results.txt > fixed_taxonomy_results.txt

13. Delete the second column, which is the column that has all taxonomic info, including ones like supergroup and subphylum

awk -F'\t' 'BEGIN {OFS="\t"} {print $1, $3}' fixed_taxonomy_results.txt > filtered_taxonomy_results.txt

14. I have made a new file called filtered_taxonomy_results.txt. Column 1 contains taxIDs and column 2 contains taxonomic info. The taxonomic levels are separated using semicolons. Now want to separate the different taxonomic levels into separate columns

awk -F'\t' 'BEGIN {OFS="\t"} {split($2, levels, ";"); print $1, levels[1], levels[2], levels[3], levels[4], levels[5], levels[6], levels[7]}' filtered_taxonomy_results.txt > separated_taxonomy_results.txt

15. Now need to combine the taxonomic info with the blast_SSU file that has sequences. Do this by matching the taxIDs between the files and merging all results into one file

awk 'NR==FNR {tax[$1] = $2"\t"$3"\t"$4"\t"$5"\t"$6"\t"$7"\t"$8; next} {print $0, tax[$NF]}' separated_taxonomy_results.txt blast_SSU_out_complete.txt> merged_results.txt

16. There are multiple results for each sequence because that’s how BLAST works. Need to have only one result per sequence. Figure out which to keep by taking the top hit. Sort results by bit score and keep only the best match.

sort -k1,1 -k12,12nr merged_results.txt | awk '!seen[$1]++' > top_hit_results.txt

17. Keep only sequence and taxonomy information

awk -F'\t' 'BEGIN {OFS="\t"} {print $13, $15, $16, $17, $18, $19, $20, $21}' top_hit_results.txt > blast_taxonomy_results.txt

18. Add a header row at the top with the following names: Sequence, Phylum, Class, Order, Family, Genus, Species

echo -e "Sequence\tPhylum\tClass\tOrder\tFamily\tGenus\tSpecies" | cat - blast_taxonomy_results.txt > blast_taxonomy_results_with_header.txt

19. Fill in empty cells with NA

awk 'BEGIN {FS=OFS="\t"} {for(i=1; i<=NF; i++) if($i == "") $i="NA"; print $0}' blast_taxonomy_results_with_header.txt > blast_taxonomy_filled.txt

20. There is an extra column at the end, after the species column. Delete this.

awk -F'\t' 'BEGIN {OFS="\t"} {NF--; print}' blast_taxonomy_filled.txt > blast_taxonomy_filled_mod.txt

21. Count how many are NA at species level:

awk -F'\t' '{if ($NF == "NA") count++} END {print count}' blast_taxonomy_filled_mod.txt

- 2,469 out of 3,943
- 62.6% are NA

22. Change blast_taxonomy_filled.txt into a csv file

tr '\t' ',' < blast_taxonomy_filled_mod.txt > blast_taxonomy_filled_mod.csv

23. Upload file from aligning ASVs to UNITE database (08_assigned_taxonomy_no_kingdom) to HPC folder
    a. This is same as other 08 file, but the kingdom column is deleted since I don’t have the kingdom column in the BLAST file
24. Combine the results from BLAST and UNITE. Merge results based on sequences. If both UNITE and BLAST have results for the same ASV, prioritise the result from UNITE
    a. Now I have a file from aligning ASVs to UNITE database (08_assigned_taxonomy_no_kingdom.csv) and the blast_taxonomy_filled_mod.csv file from blast-ing all of my ASVs. I need to combine the results from these together based on sequences. If UNITE and BLAST both have taxonomy results (i.e., not NA) for the same ASV, I want the UNITE taxonomy to be prioritised. The resultant file (call it unite_blast_taxonomy.csv) should have sequences in column 1, then phylum, class, order, family, genus, species.

awk -F',' 'NR==FNR {for (i=2; i<=NF; i++) if ($i != "NA") tax[$1, i] = $i; next}

NR>FNR {

printf "%s", $1;

for (i=2; i<=NF; i++) {

printf ",%s", ($1, i) in tax ? tax[$1, i] : $i

}

print ""

}' 08_assigned_taxonomy_no_kingdom.csv blast_taxonomy_filled_mod.csv > unite_blast_taxonomy.csv

- NR==FNR 🡪 Reads 08_assigned_taxonomy_no_kingdom.csv first and stores its taxonomy if it’s not “NA”
- NR>FNR 🡪 Reads blast_taxonomy_filled_mod.csv, filling in missing taxonomic levels on if not provided already by UNITE
- printf 🡪 Ensures all taxonomic levels are printed properly
- Final output 🡪 unite_blast_taxonomy.csv

25. Count how many are NA at species level

awk -F',' '{if($7 == "NA") count++} END {print count}' unite_blast_taxonomy.csv

- 1,936 out of 3,943 are NA aka 49%
- 1910 at genus
- 1753 at family
- 1607 at class (40.8%)

26. I tried uploading the file into R, but it says there are duplicate rows (aka duplicate sequences). I used this code to see that there are 447 duplicate rows:

data <- read.csv("G:/My Drive/ECORISC PhD/Experiments/Experiment 1-3/Sequencing analysis/ITS/blast_taxonomy_filled_mod.csv")

sum(duplicated(data$Sequence))

27. I then checked if there are duplicates on the HPC using the following code:

awk -F',' '{print $1}' top_hit_results.txt | sort | uniq -d

- No duplicates

awk -F',' '{print $1}' unite)_blast_taxonomy.csv | sort | uniq -d

- Yes duplicates. The duplicates are happening at the merging with unite step

28. Remove duplicates, keeping the first occurrence of each sequence:

awk -F',' '!seen[$1]++' unite_blast_taxonomy.csv > unite_blast_taxonomy_no_duplicates.csv

29. Now work in R on building phyloseq objects and further analysis.

**Supplementary fig. 2 Absolute amount of P (a,c,e) and N (b,d,f) in dwarf runner bean shoot tissues.** DOX = a,b; SMX = c,d; and FLUB = e,f. Control (‘No Drug’) treatments in blue, experimental (‘Drug’) treatments in orange. Whiskers extend to the 25^th^ and 75^th^ percentiles, with boxes encompassing interquartile range. Median lines exist inside each box. Significant differences are denoted as * p<0.05. ns = a non-significant difference

**Supplementary fig. 3 Alpha diversity metrics for fungi in soil (a,c,e: Shannon’s diversity; b,d,f: Simpson diversity).** DOX = a,b; SMX = c,d; FLUB = e,f. Control (‘No Drug’) treatments in blue, experimental (‘Drug’) treatments in orange. Whiskers extend to the 25^th^ and 75^th^ percentiles, with boxes encompassing interquartile range. Median lines exist inside each box. ns = a non-significant difference

**Supplementary fig. 4 Alpha diversity metrics for fungi in root (a,c,e: Shannon’s diversity; b,d,f: Simpson diversity).** DOX = a,b; SMX = c,d; FLUB = e,f. Control (‘No Drug’) treatments in blue, experimental (‘Drug’) treatments in orange. Whiskers extend to the 25^th^ and 75^th^ percentiles, with boxes encompassing interquartile range. Median lines exist inside each box. ns = a non-significant difference

**Supplementary fig. 5 Alpha diversity metrics for bacteria in root (a,c,e: Shannon’s diversity; b,d,f: Simpson diversity).** DOX = a,b; SMX = c,d; FLUB = e,f. Control (‘No Drug’) treatments in blue, experimental (‘Drug’) treatments in orange. Whiskers extend to the 25^th^ and 75^th^ percentiles, with boxes encompassing interquartile range. Median lines exist inside each box. n.s.= a non-significant difference

**Supplementary fig. 6 Beta diversity NMDS plots for fungi in roots (a,c,e) and soil (b,d,f).** DOX = a,b; SMX = c,d; FLUB = e,f. Control (‘No Drug’) treatments in blue, experimental (‘Drug’) treatments in orange. ns = a non-significant difference

**Supplementary fig. 7 Beta diversity NMDS plots for bacteria in roots (a,c,e) and soil (b,d,f).** DOX = a,b; SMX = c,d; FLUB = e,f. Control (‘No Drug’) treatments in blue, experimental (‘Drug’) treatments in orange. ns = a non-significant difference

**Supplementary fig. 8 Mean relative abundances for rhizobial families in soil (a,b,c) and roots (d,e,f) bar chart.** DOX = a,d; SMX = b,e; FLUB = c,f. Control (‘No Drug’) treatments in blue, experimental (‘Drug’) treatments in orange. Error bars represent standard deviations. n.s.= a non-significant difference

**Supplementary table 1: Welch’s t-test statistical results for shoot and root biomass (Figure 1).**

|  | **p-value** | **df** | **t** |
| --- | --- | --- | --- |
| **DOX** |  |  |  |
| Shoot biomass | 0.8835 | 24.41 | 0.1480 |
| Root biomass | 0.1223 | 18.55 | 1.619 |
| **SMX** |  |  |  |
| Shoot biomass | 0.5818 | 24.93 | 0.5580 |
| Root biomass | 0.0815 | 21.40 | 0.1828 |
| **FLUB** |  |  |  |
| Shoot biomass | 0.7365 | 22.08 | 0.3407 |
| Root biomass | 0.0750 | 26.68 | 1.853 |

**Supplementary table 2: Welch’s t-test statistical results for [total P], [total N], total P, and total N (Figure 2 and Supplementary Figure 1).**

|  | **p-value** | **df** | **t** |
| --- | --- | --- | --- |
| **DOX** |  |  |  |
| [Total P] | 0.6272 | 24.03 | 0.4919 |
| [Total N] | 0.0963 | 17.42 | 1.758 |
| Total P | 0.4688 | 25.93 | 0.7352 |
| Total N | 0.4087 | 18.14 | 0.8457 |
| **SMX** |  |  |  |
| [Total P] | 0.1049 | 24.12 | 1.685 |
| [Total N] | **0.0007** | 15.89 | 4.217 |
| Total P | 0.7467 | 24.51 | 0.3267 |
| Total N | **0.0156** | 16.18 | 2.701 |
| **FLUB** |  |  |  |
| [Total P] | 0.9437 | 27.60 | 0.07123 |
| [Total N] | 0.5385 | 22.48 | 0.6246 |
| Total P | 0.9107 | 27.88 | 0.1132 |
| Total N | 0.7573 | 27.97 | 0.3120 |

**Supplementary table 3: Welch’s t-test statistical results for root colonisation by AM fungi and soil AM hyphal density (Figure 3).**

|  | **p-value** | **df** | **t** |
| --- | --- | --- | --- |
| **DOX** |  |  |  |
| Colonisation | 0.7438 | 24.82 | 0.3304 |
| Hyphal density | **<0.0001** | 25.03 | 5.422 |
| **SMX** |  |  |  |
| Colonisation | 0.6992 | 17.47 | 0.3929 |
| Hyphal density | 0.7765 | 24.67 | 0.2870 |
| **FLUB** |  |  |  |
| Colonisation | **<0.0001** | 27.56 | 0.5390 |
| Hyphal density | 0.4831 | 23.26 | 0.7128 |

**Supplementary table 4: Mann Whitney-U test statistical results AM fungal-dwarf runner bean nutrient exchange (Figure 4).**

|  | **p-value** | **Mann-Whitney U** | **‘No Drug’ df** | **‘Drug’ df** | **‘No Drug’ median** | **‘Drug’ median** |
| --- | --- | --- | --- | --- | --- | --- |
| **DOX** |  |  |  |  |  |  |
| Shoot [^33^P] | **0.0255** | 9 | 7 | 8 | 0 | 0.0110 |
| Shoot [^15^N] | 0.2584 | 18 | 7 | 8 | 0 | 0.000337 |
| Fungal C | 0.2746 | 75 | 13 | 15 | 0 | 0.0004685 |
| **SMX** |  |  |  |  |  |  |
| Shoot [^33^P] | 0.3860 | 20 | 7 | 8 | 0.00252 | 0.000550 |
| Shoot [^15^N] | 0.7692 | 25 | 7 | 8 | 0.05410 | 0 |
| Fungal C | 0.3361 | 71 | 13 | 14 | 0.001691 | 0.001011 |
| **FLUB** |  |  |  |  |  |  |
| Shoot [^33^P] | >0.9999 | 32 | 8 | 8 | 5.6x10^-5^ | 0 |
| Shoot [^15^N] | 0.2364 | 21 | 8 | 8 | 0 | 0.03122 |
| Fungal C | **0.0417** | 65 | 15 | 14 | 0.001640 | 0.000 |

**Supplementary table 5: Welch’s t-test statistical results for fungal alpha diversity (Supplementary Figure 2,3).**

|  | **p-value** | **df** | **t** |
| --- | --- | --- | --- |
| **DOX** |  |  |  |
| Fungal soil Shannon’s diversity index | 0.7492 | 13.63 | 0.3263 |
| Fungal soil Simpson diversity index | 0.8956 | 13.94 | 0.1337 |
| Fungal root Shannon’s diversity index | 0.6080 | 10.69 | 0.5284 |
| Fungal root Simpson diversity index | 0.1950 | 10.08 | 1.388 |
| **SMX** |  |  |  |
| Fungal soil Shannon’s diversity index | 0.4718 | 13.60 | 0.7401 |
| Fungal soil Simpson diversity index | 0.4214 | 13.03 | 0.8302 |
| Fungal root Shannon’s diversity index | 0.2448 | 13.72 | 1.215 |
| Fungal root Simpson diversity index | 0.2805 | 12.44 | 1.128 |
| **FLUB** |  |  |  |
| Fungal soil Shannon’s diversity index | 0.7642 | 13.61 | 0.3060 |
| Fungal soil Simpson diversity index | 0.9894 | 13.97 | 0.01353 |
| Fungal root Shannon’s diversity index | 0.3728 | 6.694 | 0.9550 |
| Fungal root Simpson diversity index | 0.2798 | 6.803 | 1.174 |

**Supplementary table 6: Welch’s t-test statistical results for bacterial alpha diversity (Figure 7 and Supplementary Figure 4).**

|  | **p-value** | **df** | **t** |
| --- | --- | --- | --- |
| **DOX** |  |  |  |
| 16S soil Shannon’s diversity index | **0.0517** | 12.11 | 2.158 |
| 16S soil Simpson diversity index | **0.1437** | 12.33 | 1.562 |
| 16S root Shannon’s diversity index | 0.5529 | 11.13 | 0.6119 |
| 16S root Simpson diversity index | 0.6452 | 10.87 | 0.6452 |
| **SMX** |  |  |  |
| 16S soil Shannon’s diversity index | **0.1382** | 12.13 | 1.587 |
| 16S soil Simpson diversity index | **0.0850** | 10.36 | 1.904 |
| 16S root Shannon’s diversity index | 0.2877 | 11.15 | 1.117 |
| 16S root Simpson diversity index | 0.3011 | 7.905 | 1.106 |
| **FLUB** |  |  |  |
| 16S soil Shannon’s diversity index | 0.7518 | 11.67 | 0.3239 |
| 16S soil Simpson diversity index | 0.9514 | 11.96 | 0.06225 |
| 16S root Shannon’s diversity index | 0.2430 | 13.36 | 1.222 |
| 16S root Simpson diversity index | 0.2864 | 10.42 | 1.124 |

**Supplementary table 7: PERMANOVA statistical results for fungal and bacterial NMDS plots (Supplementary Figure 5,6).**

|  | **Stress** | **p-value** | **F** | **df** | **R^2^** |
| --- | --- | --- | --- | --- | --- |
| **DOX** |  |  |  |  |  |
| Fungal soil NMDS | 8.710045x10^-5^ | 0.924 | 0.2372 | 1/14 | 0.01666/0.98334 |
| Fungal root NMDS | 0.05661136 | 0.868 | 0.4686 | 1/14 | 0.03239/0.96761 |
| Bacterial soil NMDS | 0.06537545 | 0.340 | 0.9886 | 1/13 | 0.07067/0.92933 |
| Bacterial root NMDS | 7.728746 x10^-5^ | 0.886 | 0.5291 | 1/14 | 0.03642/0.96358 |
| **SMX** |  |  |  |  |  |
| Fungal soil NMDS | 0.0735053 | 0.867 | 0.3516 | 1/14 | 0.0245/0.9755 |
| Fungal root NMDS | 0.126173 | 0.250 | 1.2533 | 1/14 | 0.08216/0.91784 |
| Bacterial soil NMDS | 0.0712615 | 0.570 | 0.6752 | 1/13 | 0.04938/0.95062 |
| Bacterial root NMDS | 0.03792925 | 0.838 | 0.4634 | 1/14 | 0.03204/0.96796 |
| **FLUB** |  |  |  |  |  |
| Fungal soil NMDS | 0.05612566 | 0.902 | 0.2952 | 1/14 | 0.02065/0.97935 |
| Fungal root NMDS | 0.069334 | 0.848 | 0.4654 | 1/10 | 0.04447/0.95553 |
| Bacterial soil NMDS | 9.139791x10^-5^ | 0.355 | 1.0727 | 1/12 | 0.08206/0.91794 |
| Bacterial root NMDS | 0.03515344 | 0.967 | 0.3581 | 1/14 | 0.02494/0.97506 |

**Supplementary table 8: Welch’s t-test statistical results for mean relative abundance of the top 10 most abundant bacterial classes in soil (Figure 8).**

| **Bacterial class** | **p-value** | **df** | **t** |
| --- | --- | --- | --- |
| **DOX** |  |  |  |
| Actinobacteria | 0.098 | 10.2 | -1.82 |
| Alphaproteobacteria | **0.0363** | 8.96 | 2.46 |
| Bacilli | 0.389 | 11.4 | -0.895 |
| Bacteroidia | 0.242 | 7.42 | -1.27 |
| Clostridia | 0.182 | 8.18 | -1.46 |
| Desulfitobacteriia | 0.260 | 10.0 | -1.19 |
| Gammaproteobacteria | 0.448 | 10.2 | -0.790 |
| Limnochordia | 0.552 | 12.0 | -0.612 |
| Polyangia | 0.927 | 11.2 | -0.0932 |
| Thermoleophilia | 0.700 | 12.0 | -0.395 |
| **SMX** |  |  |  |
| Actinobacteria | 0.302 | 12.0 | -1.08 |
| Alphaproteobacteria | 0.320 | 13.0 | 1.03 |
| Bacilli | 0.935 | 8.98 | 0.0841 |
| Bacteroidia | 0.438 | 11.5 | -0.804 |
| Clostridia | 0.766 | 13.0 | -0.303 |
| Desulfitobacteriia | 0.888 | 12.0 | -0.143 |
| Gammaproteobacteria | 0.347 | 11.5 | -0.980 |
| Limnochordia | 0.681 | 13.0 | -0.421 |
| Polyangia | 0.698 | 12.7 | -0.396 |
| Thermoleophilia | 0.0856 | 8.44 | -1.95 |
| **FLUB** |  |  |  |
| Actinobacteria | 0.620 | 6.96 | 0.519 |
| Alphaproteobacteria | 0.305 | 8.04 | 1.10 |
| Bacilli | 0.443 | 6.23 | -0.818 |
| Bacteroidia | 0.296 | 7.16 | 1.13 |
| Clostridia | 0.299 | 6.03 | -1.14 |
| Desulfitobacteriia | 0.397 | 6.11 | -0.911 |
| Gammaproteobacteria | 0.405 | 6.50 | -0.891 |
| Limnochordia | 0.284 | 6.08 | -1.17 |
| Polyangia | 0.349 | 5.66 | 1.02 |
| Thermoleophilia | 0.630 | 6.77 | 0.505 |

**Supplementary table 9: Welch’s t-test statistical results for mean relative abundance of Rhizobiaceae in soil and root (Supplementary figure 8).**

|  | **p-value** | df | t |
| --- | --- | --- | --- |
| **DOX** |  |  |  |
| Rhizobiaceae soil | 0.212 | 10.8 | 1.33 |
| Rhizobiaceae root | 0.933 | 13.9 | -0.0853 |
| **SMX** |  |  |  |
| Rhizobiaceae soil | 0.850 | 12.1 | -0.194 |
| Rhizobiaceae root | 0.785 | 13.6 | -0.279 |
| **FLUB** |  |  |  |
| Rhizobiaceae soil | 0.143 | 10.7 | 1.58 |
| Rhizobiaceae root | 0.965 | 13.8 | 0.0441 |
